# The promising role of probiotics/prebiotics/synbiotics in energy metabolism biomarkers in patients with NAFLD: A systematic review and meta-analysis

**DOI:** 10.3389/fpubh.2022.862266

**Published:** 2022-07-25

**Authors:** Shudi Li, Jiangkai Liu, Zhen Wang, Fei Duan, Zi Jia, Xinju Chen, Suling Li

**Affiliations:** ^1^Henan University of Chinese Medicine, Zhengzhou, China; ^2^The First Affiliated Hospital of Henan University of TCM, Zhengzhou, China

**Keywords:** NAFLD, probiotics, prebiotics, synbiotics, microbiota modulation, energy metabolism biomarkers

## Abstract

**Background:**

Nonalcoholic fatty liver disease (NAFLD) is a chronic liver disease with a high prevalence worldwide, seriously harming human health, and its pathogenesis remains unclear. In recent years, increasing evidence has indicated that intestinal microbiota plays an important role in the occurrence and development of NAFLD. The regulation method of probiotics/prebiotics/synbiotics can alter the intestinal microbiota and has been suggested as an option in the treatment of NAFLD.

**Methods:**

Five databases of PubMed, Embase, the Cochrane Library, clinicaltrails.gov, and China National Knowledge Infrastructure were searched initially, and then the eligible studies were screened. Finally, the data of included studieswere extracted, combined and analyzed

**Results:**

A total of 29 randomized controlled trials involving 2,110 patients were included in this study. The results showed that using probiotics/prebiotics/synbiotics in the intervention group could reduce the levels of glucose (SMD = −0.23, 95% CI [−0.45, −0.01], *P* = 0.04), HOMA-IR (SMD = −0.47, 95% CI [−0.63, −0.31], *P* < 0.00001) and insulin (SMD = −0.46, 95% CI [−0.76, −0.16], *P* = 0.002) in sugar metabolism; in terms of lipid metabolism, the levels of TC (SMD = −0.62, 95%CI [−0.87, −0.36], *P* < 0.00001), and LDL-C (SMD = −0.57, 95%CI [−0.85, −0.28], *P* < 0.00001) were decreased; and the level of ALB was decreased in protein metabolism (SMD = −0.34, 95%CI [−0.61, −0.06], *P* = 0.02).

**Conclusions:**

Based on the current evidence, probiotics/prebiotics/synbiotics may improve energy metabolism biomarkers in the NAFLD population, but these effects still need to be confirmed by further research.

**Systematic Review Registration:**

https://www.crd.york.ac.uk/PROSPERO/#aboutpage.

## Introduction

Nonalcoholic fatty liver disease (NAFLD) is a chronic liver disease characterized by excessive accumulation of fat in liver cells (1). It includes a wide range of pathological liver diseases, from simple accumulation of liver fat to nonalcoholic steatohepatitis (NASH) with or without fibrosis, which can eventually progress to cirrhosis and liver cancer (2). The global prevalence of NAFLD has reached 25.2%, posing a serious threat to human health (3). The pathogenesis of NAFLD is still unclear, and the widely accepted concept of “multiple strikes” highlights the important role of various genetic and environmental factors in the multifactorial pathogenesis of NAFLD (4). Currently, no effective drugs for NAFLD have been approved (5).

The intestinal microbiota is a collective term for a large number of microorganisms existing in the human intestinal tract, whose metabolic activities can affect nutrient absorption and energy homeostasis (6). Recent studies have shown that intestinal microbiota plays a vital role in the development and progression of NAFLD, including regulating energy homeostasis by increasing carbohydrate fermentation to short-chain fatty acids, and activating the resynthesis of triglycerides and bacterial-derived toxins in the liver (7–10). The interaction between the liver and intestine (gut-liver axis) is not only involved in the pathogenesis of NAFLD, but also may be an important factor leading to the progression of NAFLD to NASH and related liver fibrosis (11, 12).

Probiotics, prebiotics, and synbiotics (PPS) have been proved to be therapeutic methods that can change the composition of the microbiota and restore microbial balance (13). Probiotics are living non-pathogenic microorganisms, prebiotics are defined as indigestible fiber compounds, and synbiotics are combinations of probiotics and prebiotics. They can cause specific changes in the composition and activity of the gastrointestinal microbiota when ingested, and increase the secretion of endogenous intestinal nutrient peptides (14). Studies have shown that PPS can regulate the composition of intestinal flora and the production of antibacterial factors, change the permeability and function of intestinal epithelial cells and reduce the permeability of intestinal endotoxin to affect the occurrence and development of NAFLD. They can also exert effects on NAFLD by modifying endotoxemia, inhibiting inflammatory response, and regulating the immune system (15–17).The PPS regulation method has been suggested as a treatment for NAFLD (18), and a number of randomized controlled trials have been conducted in clinical practice to confirm that PPS regulation can improve NAFLD. However, most studies have focused on the improvement of liver function and inflammatory indicators in NAFLD by the PPS regulation method, while there is a lack of systematic conclusions on the improvement effect of NAFLD on energy metabolism biomarkers. Based on this, this study adopts the method of meta-analysis to systematically evaluate PPS regulation on the energy metabolism biomarkers in NAFLD patients, in order to provide a reference and basis for the clinical practice of NAFLD.

## Materials and methods

The present study was conducted in accordance with the Preferred Reporting Items for Systemic Reviews and Meta-Analysis (PRISMA) statement to ensure transparent reporting of the scientific evidence (19), and was registered with PROSPERO in advance (CRD:42021288543, https://www.crd.york.ac.uk/PROSPERO/).

### Inclusion and exclusion criteria

The included clinical studies met the following criteria: clinical trials were randomized controlled trials; participants were patients with NAFLD, regardless of age, gender, or race; the interventions were probiotics and/or prebiotics and/or synbiotics; except for the intervention method, the treatment of the control group was the same as that of the intervention group; the outcome measures included glucose (Glu), insulin, homeostatic model assessment of IR (HOMA-IR), total cholesterol (TC), triglycerides (TG), high-density lipoprotein cholesterol (HDL-C), low-density lipoprotein cholesterol (LDL-C), albumin.

Exclusion criteria: patients with alcoholic steatohepatitis, alcoholic fatty liver, cirrhosis or liver cancer; patients receiving additional medication or genetic predisposition (single nucleotide polymorphisms); liver transplant patients; conference papers or abstracts; non-original research or case reports; and non-peer-reviewed articles.

### Database resources

We searched databases including PubMed, Embase, the Cochrane Library, clinicaltrails.gov, China National Knowledge Infrastructure.

### Search strategy

Related terms of “probiotics”, “prebiotics”, “synbiotics” and “nonalcoholic fatty liver disease” were used to search the above database. The search and selection period was from January 1, 2000 to September 31, 2021. The search strategies and results are detailed in the Appendix (see in Supplementary Table S1).

### Study selection protocol

The search and selection of studies were performed by three professionally trained researchers who were informed of inclusion and exclusion criteria prior to searching and selection. Studies were firstly screened by title and abstract, followed by further assessment of the full text based on inclusion and exclusion criteria. Finally, the screened full texts were compared. The three researchers negotiated and voted to resolve disagreements and discrepancies.

### Data synthesis and analysis

The statistical analysis was carried out using statistical software RevMan Version 5.3 (The Cochrane Collaboration, Copenhagen, Denmark) and the standard mean difference (SMD) synthesis was used uniformly since the outcome indicators included in this study were all continuous values. If the data were available and sufficient, we would also conduct subgroup analyses, based primarily on the type of diease (NAFLD/NASH), type of intervention (probiotic, prebiotic, synbiotic), type of control (placebo/non placebo), administratingduration ( ≤ 12 weeks; >12 weeks, <24 weeks; ≥24 weeks). *I*^2^ test was used to assess the magnitude of heterogeneity. The fixed-effects model was used only when the *I*^2^ value was <50%; otherwise, the random-effects model was used. The sensitivity analysis and subgroup analysis would be performed to define the source of heterogeneity. Any referenced statistic adopted a two-sided test, and the significance level was set as 0.05.

### Risk of bias and study quality assessment

Two authors independently evaluated the titles and abstracts of available articles to exclude irrelevant studies. Full texts of selected articles were assessed individually for eligibility on the basis of the above inclusion criteria. The Cochrane Collaboration's Risk of Bias tool was used to assess bias in the eligible RCTs. Sensitivity analysis was conducted by deleting the included studies in sequence to identify the stability of the total effect. If there were more than 10 articles for a certain outcome index, a funnel plotwas used to analyze whether publication bias existed. And Egger's test was conducted to qualify the publication bias, trim-and-fill method was applied to test how much influence the publicationbias would make on the pooling estimates.

## Results

### Study selection

**Figure 1:**
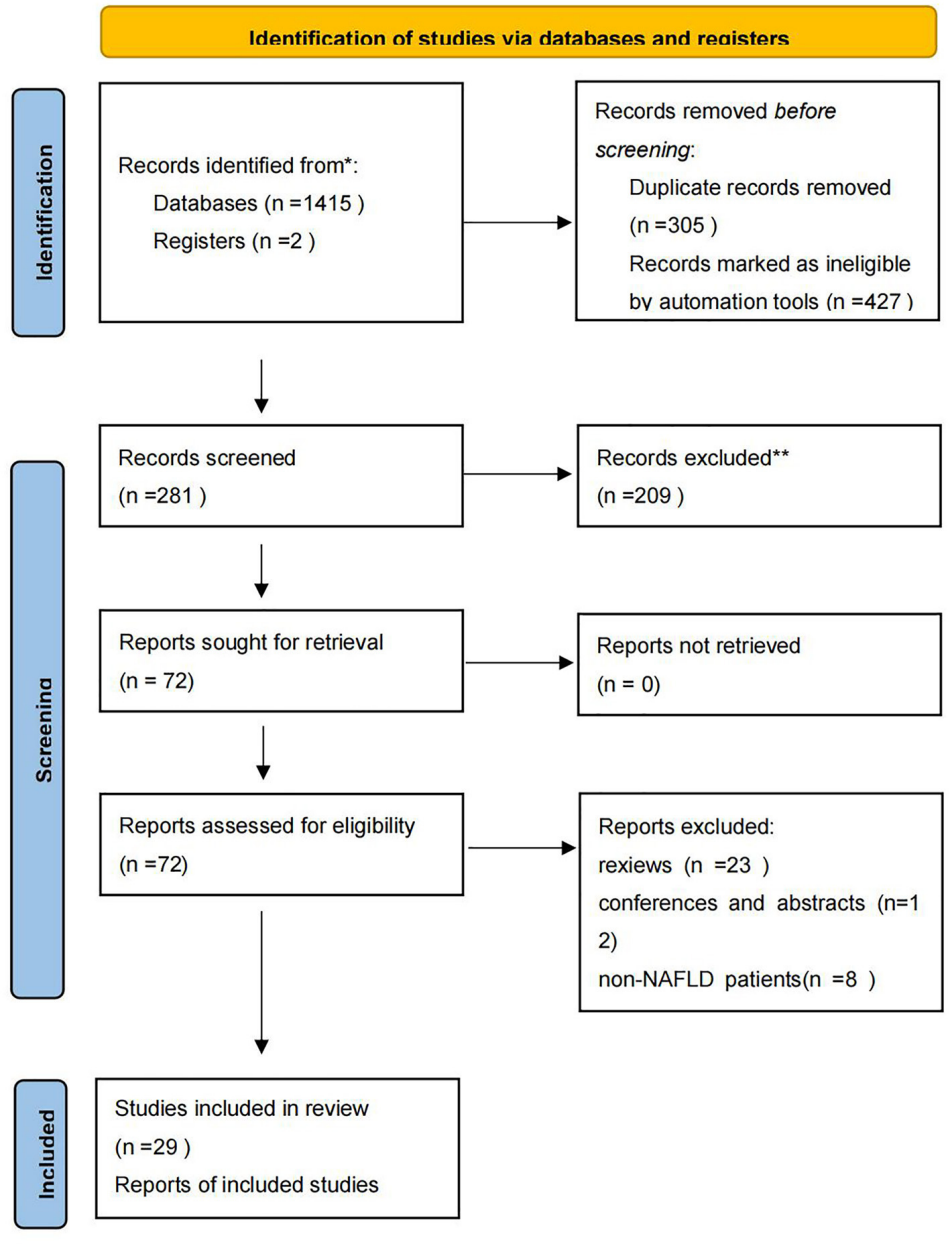
PRISMA flow diagram.

According to the formulated strategy, 1,415 articles were preliminarily retrieved up to November 2, 2021, and 29 RCTs were finally included, as shown in Figure 1.

### Basic characteristics and assessment of study quality

**Figure 2:**
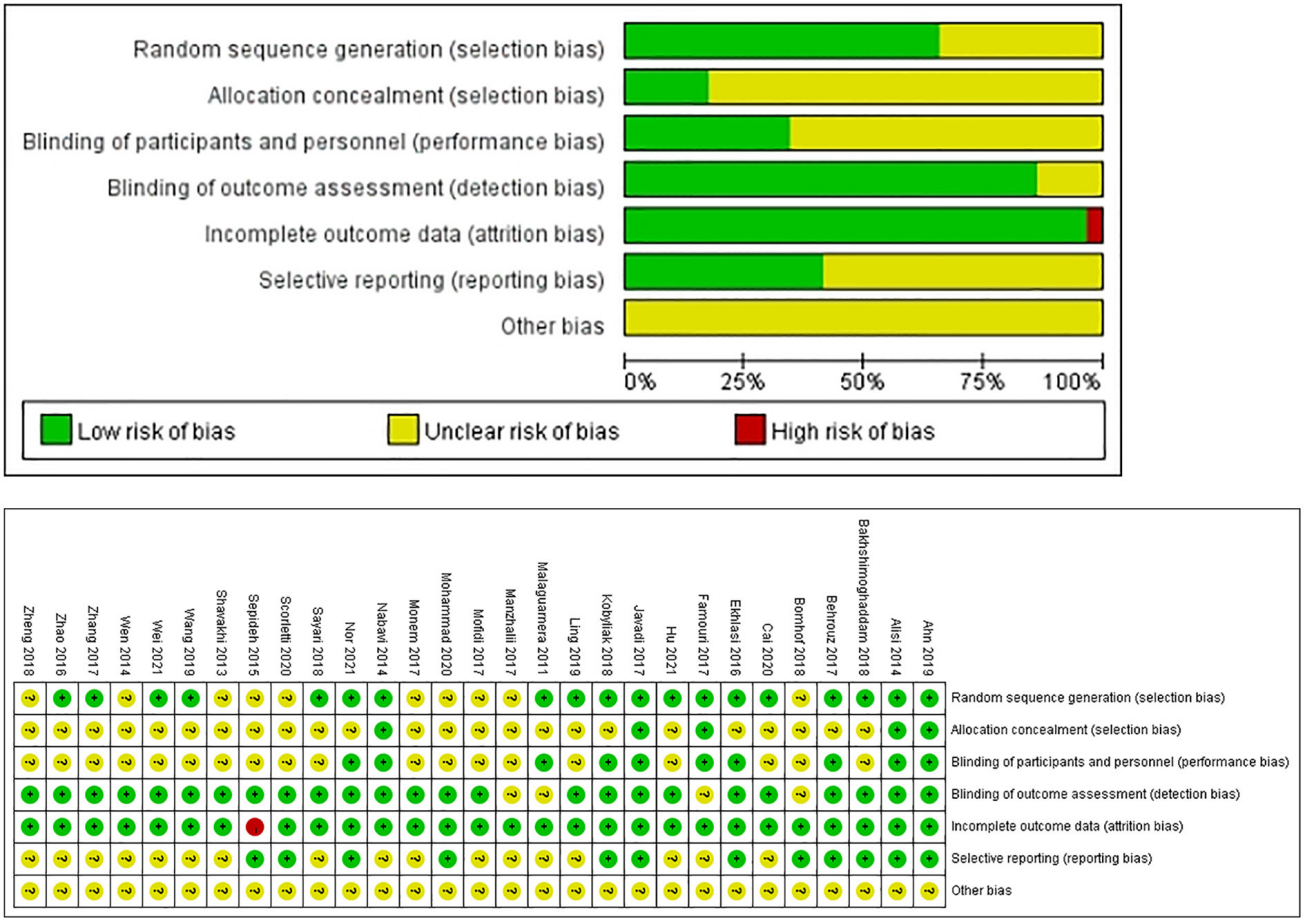
Risk-of-bias graph.

Among the 29 included studies (20–48), 20 of them evaluated probiotics, 3 evaluated prebiotics, and 9 evaluated synbiotics, with a total of 2,110 patients enrolled. The characteristics of the included studies are shown in Table 1. Subgroup analysis was performed according to the preset grouping basis and no difference was found except for the subgroups of LDL-C disease. The risk of bias assessment was carried out employing the tool provided by the Cochrane Collaboration, and the results showed that the overall risk of the included studies was medium and low, as shown in Figure 2.

**Table 1:** Characteristics of the included studies.

**Reference**	**Study type**	***E*** **/** ***C***	***m*****/*****f*** **(*****E*****;** ***C*****)**	**Age (** ***E*** **/** ***C*** **)**	**BMI (** ***E*** **/** ***C*** **)**	**Disease**	**Duration (weeks)**	**Outcomes (effect size)**
Abdel Monem (20)	RCT	15/15	9/6 8/7	44.20 ± 5.51 44.33 ± 5.62	32.56 ± 1.19 33.05 ±1.27	NASH	4	ALB(−0.09[−0.80,0.03])
	Detail of Intervention and Control: PRO(*acidophilus, tid*) +ALS vs. ALS							
Ahn et al. (21)	RCT-DB	30/35	15/17 18/18	41.7 ± 12.49 44.71 ± 13.31	30.05 ± 28.8 30.11 ± 29.1	NAFLD	12	TC(−0.11[−0.59,0.38]), TG(−0.39[−0.88,0.11]), HDL-C(−0.03[−0.52,0.46]), HOMA-IR(−0.80[−1.31,−0.29]), Insulin(−0.29[−0.78,0.20]), Glucose(−0.18[−0.67,0.31])
	Detail of Intervention and Control: PRO(*acidophilus CBTLA1, L. rhamnosus*							
	*CBT LR5, L.paracaseiCBT LPC5,P. pentosaceus CBTSL4,B. lactis CBTBL3,B.breve CBTBR3, qd*) vs. Placebo							
Alisi et al. (22)	RCT-DB	22/22	10/12 14/8	NA	27.3/25.6	NAFLD	16	TG(0.18[−0.42,0.77])
	Detail of Intervention and Control: PRO(VSL#3, 1-sachet, *qd*) vs. Placebo							
Bakhshimoghaddam et al. (23)	RCT	32/28	17/17 17/17	38.8 ± 9.0 41.1 ± 8.5	30.5 ± 4.6 31.3 ± 5.1 31.9 ± 5.1	NAFLD	24	HOMA-IR(−0.66[−1.18,−0.14]), Insulin(−0.61[−1.13,−0.09])
	Detail of Intervention and Control: SYN(lactisBB+inulin,300g, *qd*)+ADL vs. ADL							
Behrouz et al. (24)	RCT-DB	30 29 30	22/8 20/9 21/9	38.46 ± 7.11 38.41 ± 9.21 38.42 ± 10.09	29.56 ± 2.54 30.81 ± 4.74 31.90 ± 5.04	NAFLD	12	Insulin: Pre vs. Placebo: −0.44[−0.96,0.77] Pro vs. Placebo: −0.48[−1.00,0.04] HOMA-IR: Pre vs. Placebo: −0.22[−0.73,0.29] Pro vs. Placebo: −0.27[−0.78,0.25] Glucose: Pre vs. Placebo: −0.03[−0.54,0.47] Pro vs. Placebo: −0.19[−0.70,0.32]
	Detail of Intervention and Control: PRO(*Lactobacillus casei, Lactobacillus rhamnosus, Lactobacillus acidophilus, Bifidobacterium longum, Bifidobacterium breve, bid*)/PRE(Orafti P95, *bid*) vs. Placebo							
Bomhof et al. (25)	RCT	8/6	5/3 3/3	45.3 ± 5.6 53.3 ± 4.8	33.7 ± 3.0 34.8 ± 2.2	NASH	36	Glucose: 0.20[−0.86,1.26] Insulin: −0.57[−1.66,0.51]
	Detail of Intervention and Control: PRE(Orafti P95,8g,bid) vs. Placebo							
Cai et al. (26)	RCT	70/70	46/24 39/31	46.13 ± 12.72 49.62 ± 9.08	31.28 ± 3.62 30.73 ± 3.47	NAFLD	12	TC: −0.60[−0.94,−0.26] TG: −0.42[−0.75,−0.08] LDL-C: −0.30[−0.70,−0.03] HDL-C: 0.00[−0.33,0.33] HOMA-IR: −0.50[−0.83,−0.16]
	Detail of Intervention and Control: PRO(Live Combined *Bifidobacterium, Lactobacillus, Enterococcus* Powder, 1g, *bid*)+ADL vs. ADL							
Ekhlasi et al. (27)	RCT-DB	15/15	NA	NA	27.28 ± 2.21/ 27.84 ± 1.96	NAFLD	8	Glucose: −2.39[-3.36,−1.43] HOMA-IR: 0.06[-065,0.78] TG: −0.84[−1.59,−0.09] TC: −2.85[-3.90,−1.79] HDL-C: 0.27[−0.45,0.99] LDL-C: −2.61[-3.62,−1.61] Insulin: −2.25[-3.21,−1.31]
	Detail of Intervention and Control: SYN(PRO(*Lactobacillus casei, Lactobacillusrhamnosus, Streptococcusthermophilus, Bifidobacterium breve*,							
	*Lactobacillus acidophilus, Bifidobacterium longum, Lactobacillus bulgaricus*)+PRE (*fructooligosaccharide*),2 capsule, *qd*) vs. placebo							
Famouri et al. (28)	RCT-DB	32/32	NA	12.7 ± 2.2 12.6 ± 1.7	26.4 ± 4.3 26.61 ± 2.26	NAFLD	12	TC: −0.23[−0.72,0.26] HDL-C: −0.14[−0.63,0.35] LDL-C: −0.25[−0.74,0.24] TG: −0.21[−0.70,−0.28]
	Detail of Intervention and Control: PRO(*Lactobacillus acidophilus, Bifidobacterium lactis, Bifidobacterium bifidum, Lactobacillus rhamnosus,* 1 capsule, *qd*) vs. Placebo							
Javadi et al. (29)	RCT-DB	20 19 17 19	17/3 16/3 14/3 13/6	43.90 ± 9.02 38.68 ± 10 43.24 ± 6.95 42.21 ± 9.11	29.91 ± 3.88 30.96 ± 4.39 32.30 ± 4.78 30.38 ± 2.88	NAFLD	12	ALB: Pre vs. Placebo: −0.64[−1.29,−0.01] Pro vs. Placebo: −0.67[−1.33,−0.02] Syn vs. Placebo: −0.52[−1.19,0.15])
	Detail of Intervention and Control: PRO((*Bifidobacterium longum, Lactobacillus acidophilus*)/PRE(inulin HP)/SYN(PRO+PRE), 500mg, *qd*) vs. Placebo							
Kobyliak et al. (30)	RCT-DB	30/28	NA	53.4 ± 9.55 57.29 ± 10.45	34.82 ± 6.84 34.26 ± 6.17	NAFLD	8	TC: −0.01[−0.53,0.50] TG: 0.48[−0.04,1.00] HDL-C: 0.00[−0.52,0.52], LDL-C; −0.04[−0.56,0.47]
	Detail of Intervention and Control: PRO(*Lactobacillus, Lactococcus, Bifidobacteriu, Propionibacterium, Acetobacter,10g, qd*) vs. Placebo							
Malaguarnera et al. (31)	RCT-DB	34/32	18/16 15/17	46.9 ± 5.4 46.7 ± 5.7	27.3 ± 1.36 27.2 ± 1.32	NAFLD	24	TC: −0.44[−0.92,0.05] HDL-C: 0.15[−0.34,0.63] LDL-C: −0.93[−1.43,−0.42] TG: −0.40[−0.89,0.09] HOMA-IR: −0.89[−1.40,−0.38] ALB: 0.00[−0.48,0.48] Insulin: −0.42[−0.91,0.06] Glucose: 0.04[−0.44,0.53]
	Detail of Intervention and Control: SYN(*Bifidobacterium longum +*Fos, 10g, *qd*) vs. Placebo							
Manzhalii et al. (32)	RCT	38/37	11/27 16/21	43.5 ± 1.3 44.3 ± 1.5	26.4 ± 0.8 26.6 ± 0.7	NASH	12	TC: −4.45[-5.31,-3.59] TG: −0.20[−0.65,0.26] Glucose: −0.21[−0.66,0.25]
	Detail of Intervention and Control: SYN(LBSF*, qd*) +ALS vs. ALS							
Mofidi et al. (33)	RCT-DB	21/21	11/10 12/9	40.09 ± 11.44 44.61 ± 10.12	23.17 ± 1.0123.20 ± 1.07	NAFLD	28	TC: −0.71[−1.33,−0.08] TG: −0.66[−1.28,−0.44] Glucose: −0.21[−0.66,0.25] LDL-C: −0.29[−0.90,0.32] HDL-C: 0.46[−0.15,1.07] HOMA-IR: 1.68[0.97,2.39] Insulin: 0.05[−0.65,0.56]
	Detail of Intervention and Control: SYN (*Lactobacillus casei, Lactobacillus rhamnosus, Streptococcus thermophilus, Bifidobacterium breve, Lactobacillus acidophilus*,							
	*Bifidobacterium longum and Lactobacillus bulgaricus+*Fos, 1s, *bid*) vs. Placebo							
Mohamad Nor et al. (34)	RCT-DB	17/22	11/6 17/5	54.70 ± 10.19 52.47 ± 16.73	31.33 ± 12.02 28.30 ± 3.90	NAFLD	24	TC: 0.17[−0.46,0.81] TG: −0.04[−0.67,0.59] Glucose: 0.67[0.02,1.32]
	Detail of Intervention and Control: PRO(MCP® BCMC®*,bid*) vs. Placebo							
Mohammad Sadrkabir et al. (35)	RCT	33/28	NA	43.26 ± 11.42 43.72 ± 10.76	31.87 ± 5.4 30.83 ± 4.6	NAFLD	8	TC: −0.59[−1.10,−0.07] TG: 0.28[−0.23,0.78] Glucose: 0.06[−0.45,0.56] LDL-C: −0.24[−0.75,0.26] HDL-C: −0.28[−0.79,0.22]
	Detail of Intervention and Control: PRO(GeriLact, *500mg, bid*) vs. Placebo							
Nabavi et al. (36)	RCT-DB	36/36	17/19 18/18	42.75 ± 8.72 44.05 ± 8.14	30.1 ± 3.61 31.4 ± 3.6	NAFLD	8	TC: −0.75[−1.23,−0.27] TG: −0.41[−0.87,0.08] HDL-C: 0.13[−0.60,0.33] LDL-C: −0.63[−1.11,−0.16] Glucose: −0.32[−0.78,0.15]
	Detail of Intervention and Control: PRO yogurt(*B.lactis* Bb12, *L.acidophilus* La5, 500g*, qd*) vs. Conventional yogurt							
Sayari et al. (37)	RCT	70/68	NA	42.48 ± 11.41 43.42 ± 11.65	29.72 ± 3.62 29.54 ± 3.71	NAFLD	16	TC: −0.56[−0.90,−0.22] TG: 0.20[−0.13,0.54] HDL-C: (0.24[−0.10,0.57] LDL-C: −0.95[−1.30,−0.60] Glucose: −0.60[−0.95,−0.26]
	Detail of Intervention and Control: SYN(*Lactobacillus casei, Lactobacillus rhamnosus, Lactobacillus acidophilus, Lactobacillus bulgaricus, Bifidobacterium breve, Bifidobacterium*							
	*longum, Streptococcus thermophilus*+FOS, *500mg, qd*)+Sitagliptin vs. Placebo+Sitagliptin							
Scorletti et al. (38)	RCT-DB	45/44	NA	50.2 ± 12.4 51.6 ± 13.1	32.9 ± 5.5 33.2 ± 4.9	NAFLD	24	TC: 0.13[−0.28,0.55] TG: −0.21[−0.62,0.21] HDL-C: 0.03[−0.38,0.45] LDL-C: −23[−0.64,0.19] Insulin: 0.11[−0.31,0.52] Glucose: −0.04[−0.45,0.38]
	Detail of Intervention and Control: SYN(*Bifidobacterium animalis subsp. lactis* BB-12+FOS, *qd*) vs. Placebo							
Sepideh et al. (39)	RCT-DB	21/21	13/8 15/6	42.10 ± 1.99 47.33 ± 2.53	30.34 ± 1.17 29.50 ± 0.84	NAFLD	8	Glucose(−0.46[−1.07,0.15]), HOMA-IR(−0.58[−1.20,0.04]), Insulin(−0.57[−1.18,0.05])
	Detail of Intervention and Control: PRO(*Lactobacillus Casei,Lactobacillus acidophilus,Lactobacillus rhamnosus,Lactobacillus bulgaricus,Bifidobacterium breve,Bifidobacterium*							
	*breve,Bifidobacterium longum,and Streptococcus, qd*) vs. Placebo							
Shavakhi et al. (40)	RCT-DB	31/32	NA	NA	28.6 ± 2.0 28.2 ± 2.5	NASH	24	Glucose: −0.20[−0.70,0.29] TG: −0.68[−1.19,−0.17] TC: −0.43[−0.93,0.07]
	Detail of Intervention and Control: SYN(*Lactobacillus acidophilus,Lactobacillus Casei,Lactobacillus rhamnosus,Lactobacillus bulgaricus,Bifidobacterium*							
	*breve,Bifidobacterium Longum,Streptococcus thermophilus+FOS, qd*) +Metformin vs. Placebo+Metformin							
Hu et al. (41)	RCT	36/36	24/12 26/10	47.62 ± 16.41 49.56 ± 19.44	26.07 ± 3.7425.81 ± 3.52	NAFLD	12	TC: −0.50[−0.97,−0.03] TG: 3.80[3.01,4.59]
	Detail of Intervention and Control: PRO(*Bifidobacterium Lactobacillus,* 2000mg*, tid*) +ALS vs. ALS							
Ling et al. (42)	RCT	35/35	17/18 20/15	41.6 ± 16.4 41.8 ± 15.3	NA	NAFLD	16	TC: 0.02[−0.45,0.49] TG: −0.82[−1.31,−0.33] HDL-C: −0.15[−0.62,0.32] LDL-C: 0.20[−0.27,0.67] Glucose: 0.08[−0.39,0.55]
	Detail of Intervention and Control: PRO(*Bifidobacterium Lactobacillus,* 420mg*, Bid*) +UDCA vs. UDCA							
Wang et al. (43)	RCT	60/60	NA	NA	NA	NAFLD	12	TC: −0.27[−0.63,0.09] TG: 1.05[0.66,1.43] LDL-C: −0.05[−0.40,0.31] HDL-C: 0.27[−0.09,0.63] HOMA-IR: −0.28[−0.64,0.08]
	Detail of Intervention and Control: PRO(*Bifidobacterium Lactobacillus,* 1000mg*, qd*) +ALS vs. ALS							
Wei (44)	RCT	51/51	33/18 31/20	47.64/46.83	NA	NAFLD	12	TC: −0.60[−0.99,−0.20] TG: −0.66[−1.06,−0.27] HDL-C: 0.47[0.08,0.87] LDL-C; −0.82[−1.22,−0.41]
	Detail of Intervention and Control: PRO(*Bifidobacterium Lactobacillus,* 1000mg*, tid*) +GSH vs. GSH							
Wen et al. (45)	RCT	40/40	27/13 25/15	39.58 ± 11.52 40.45 ± 10.93	NA	NASH	24	TC: −1.03[−1.50,−0.56] TG: −3.68[-4.41,−2.95] HDL-C: −1.07[−1.54,−0.60] LDL-C: −1.58[−2.09,−1.08]
	Detail of Intervention and Control: PRO(*Bifidobacterium Lactobacillus, qd*) +PPC vs. PPC							
Zhang (46)	RCT	60/60	52/8 50/10	75.3 ± 10.2 74.8 ± 11.3	23.56 ± 2.6823.74 ± 2.41	NAFLD	12	TC: −0.18[−0.54,0.18] TG: −0.29[−0.65,0.07]
	Detail of Intervention and Control: PRO(*Bifidobacterium,Lactobacillus,StreptococcusThermophilus*, 4s, ti*d*)+Conventional treatment vs. Conventional treatment							
Zhao and Lv (47)	RCT-DB	33/30	20/13 18/12	44.05 ± 11.06 43.14 ± 9.57	30.38 ± 3.14 29.54 ± 2.98	NAFLD	24	TC: −0.53[−1.04,−0.03] TG: −0.63[−1.14,−0.12]
	Detail of Intervention and Control: PRO(*Bifidobacterium Lactobacillus*, 210mg, *bid*) vs. Placebo							

### Effect on sugar metabolism

A meta-analysis was carried out on the three glucose metabolism indicators of Glucose, HOMA-IR, and Insulin. And we also conduct subgroup analyses, based primarily on the types of diease (NAFLD/NASH), types of intervention (probiotic, prebiotic, synbiotic), type of control (placebo/non placebo), administrating duration ( ≤ 12 weeks; >12 weeks, <24 weeks; ≥24 weeks), but no significant difference was found between subgroups (see in Supplementary Figures S1–S11).

#### Glucose

**Figure 3:**
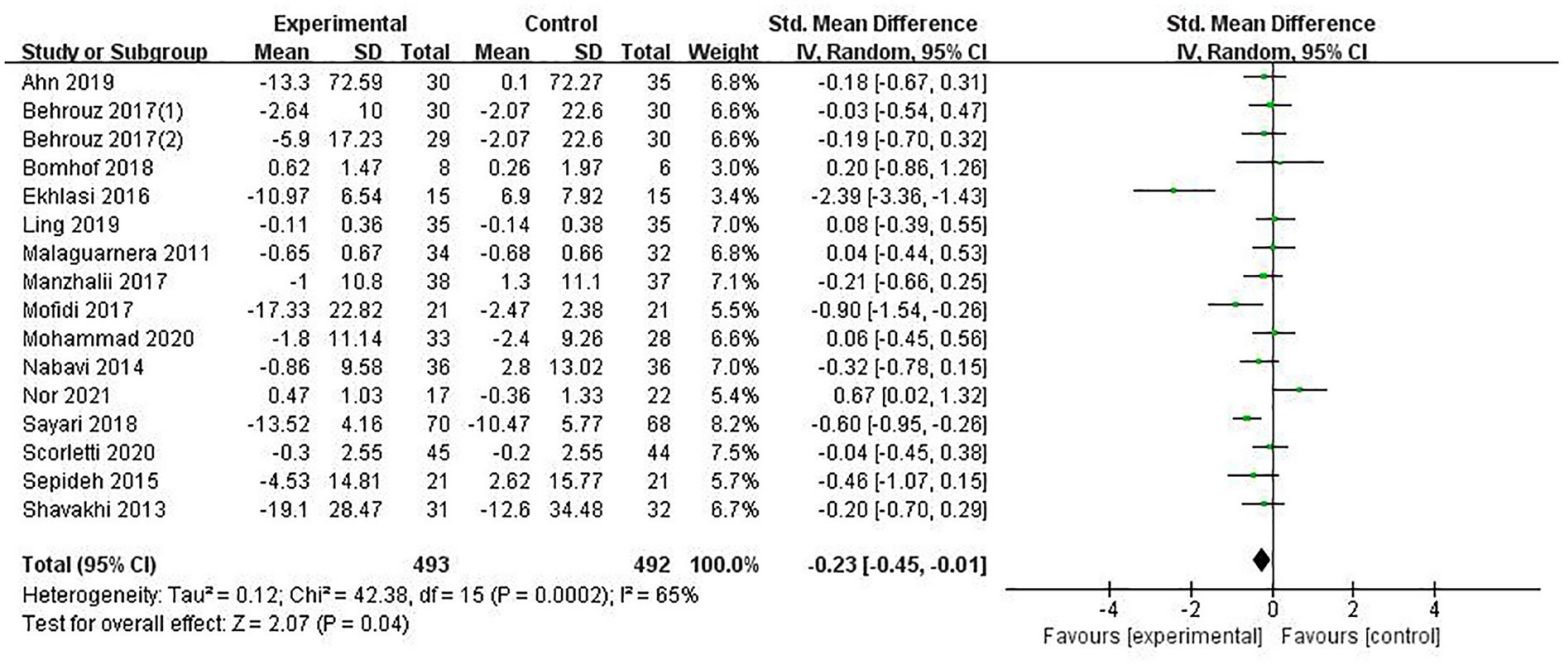
The role of PPS in Glucose in patients with NAFLD.

Glucose was reported in 15 studies involving 985 patients. The combined estimated value was SMD = −0.23 (95% CI [−0.45, −0.01], *P* = 0.04, *I*^2^ = 65%, random-effects model), and the difference was statistically significant, suggesting that the PPS regulation method could reduce glucose in NAFLD patients, as shown in Figure 3.

#### HOMA-IR

**Figure 4:**
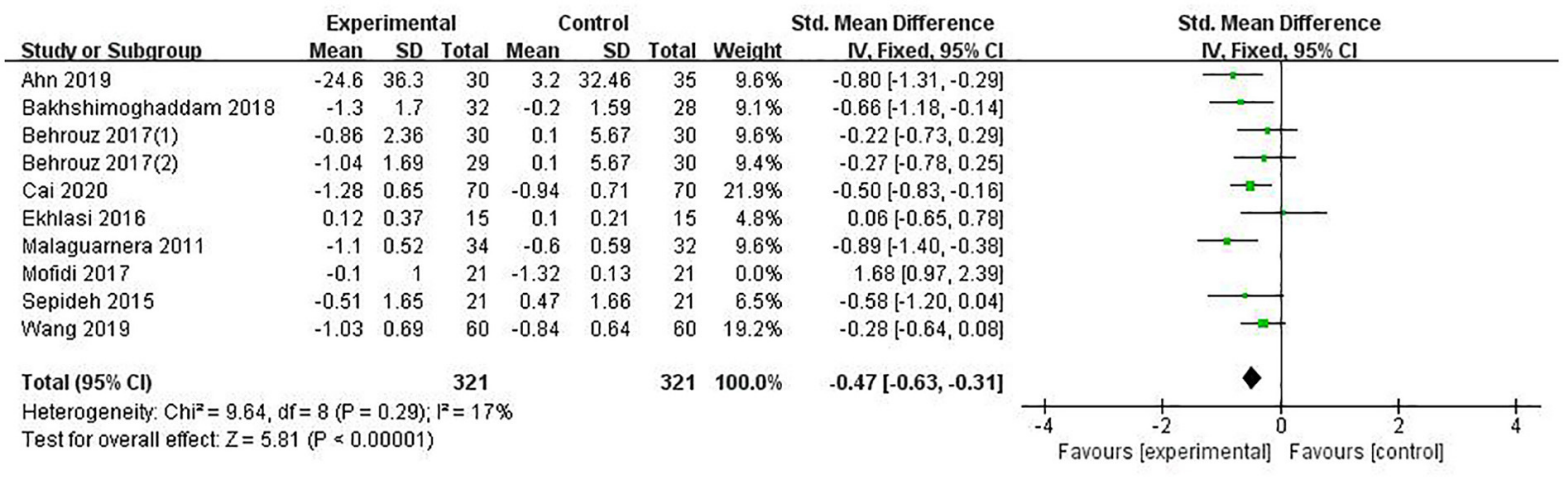
The role of PPS in HOMA-IR in patients with NAFLD.

HOMA-IR was reported in 9 studies involving 684 patients. The combined estimate was SMD = −0.29, (95% CI [−0.63, −0.06], *P* = 0.11, *I*^2^ = 79%, random-effects model). Sensitivity analysis showed that the study of Mofidi (32) affected the robustness of the pooled estimates, and after excluding this study, the results showed SMD = −0.47 (95% CI [−0.63, −0.31], *P* < 0.00001, *I*^2^ = 17 %, fixed-effects model), with a statistically significant difference. The heterogeneity of this study was analyzed, and it was considered that due to the normal range of BMI (23.17 ± 1.01/23.20 ± 1.07), the included population of the study were NAFLD patients with normal BMI and not accompanied by overweight, while the other studies concerning this indicator all included overweight NAFLD patients with BMI above the normal range. It was indicated that PPS regulation could reduce HOMA-IR in NAFLD patients, as shown in Figure 4.

#### Insulin

**Figure 5:**
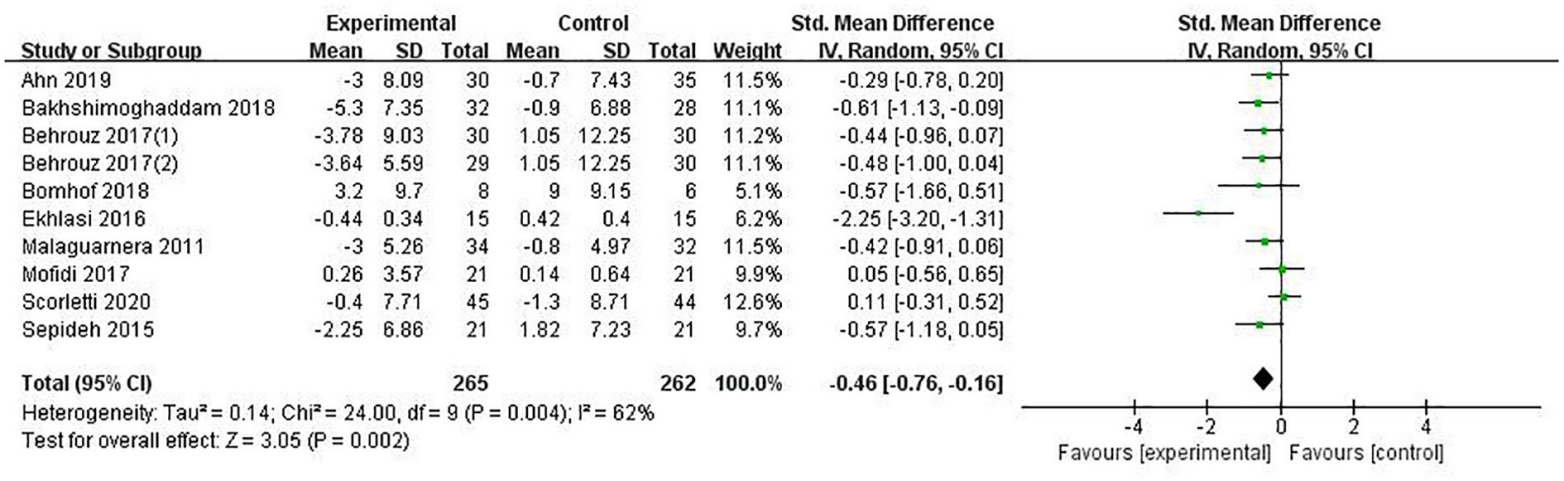
The role of PPS in Insulin in patients with NAFLD.

Insulin was reported in 9 studies involving 527 patients, with a combined estimate of SMD = −0.46 (95%CI [−0.76, −0.16], *P* = 0.002, *I*^2^ = 62%, random-effects model), and the differences were statistically significant, suggesting that PPS regulation could reduce insulin in NAFLD patients (Figure 5).

### Effect on lipid metabolism

A meta-analysis was carried out on the four lipid metabolism indicators of TC,TG,HDL-C,LDL-C. And we also conduct subgroup analyses, based primarily on the types of disease (NAFLD/NASH), types of intervention (probiotic, prebiotic, synbiotic), type of control (placebo/non placebo), administrating duration ( ≤ 12 weeks; >12 weeks, <24 weeks; ≥24 weeks). Only in the LDL-C, a statistically significant difference was found between subgroups according to disease types, and no significant difference was found in others (see in Supplementary Figures S12–S27).

#### TC

**Figure 6:**
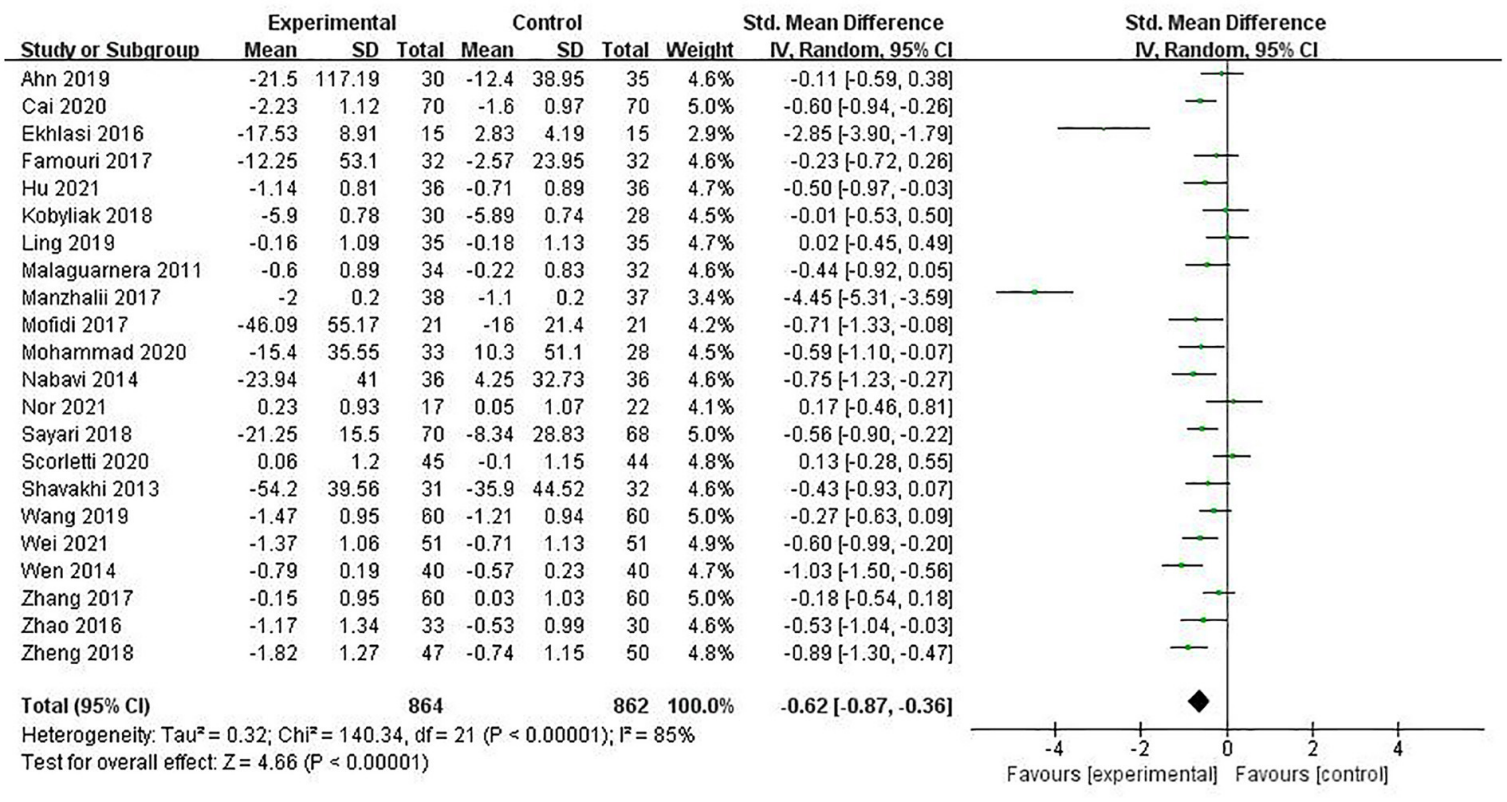
The role of PPS in TC in patients with NAFLD.

TC was reported in 22 studies involving 1,726 patients, with a combined estimated value of SMD = −0.62 (95% CI [−0.87, −0.36], *P* < 0.00001, *I*^2^ = 85%, random-effect model), indicating that PPS regulation could reduce TC in NAFLD patients, as shown in Figure 6.

#### TG

**Figure 7:**
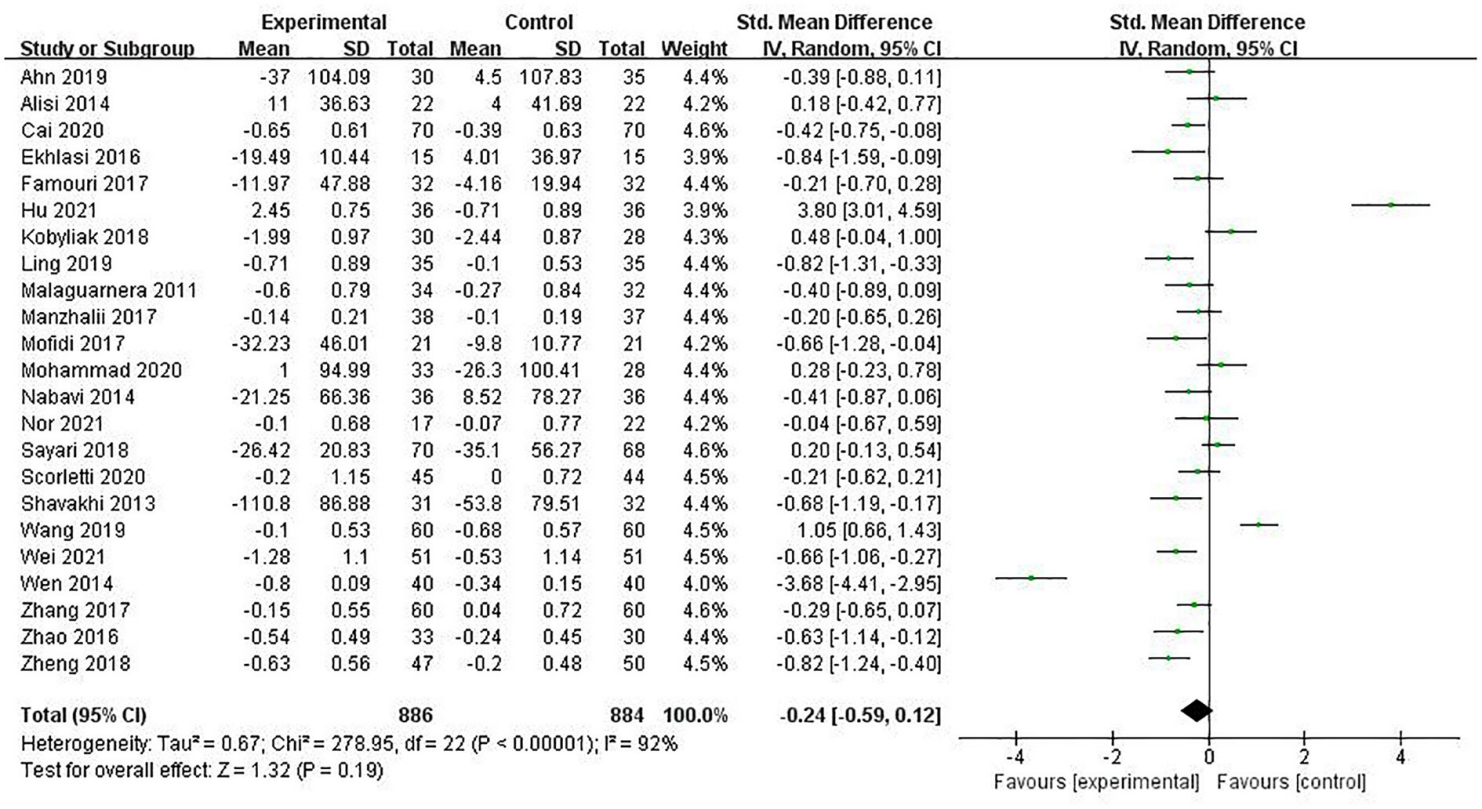
The role of PPS in TG in patients with NAFLD.

A total of 23 studies involving 1,770 patients reported the indicator of TG, and the pooled estimate was SMD = −0.24 (95% CI [−0.59, −0.12], *P* = 0.19, *I*^2^ = 92%, random-effects model). The difference was not statistically significant, as shown in Figure 7.

#### HDL-C

**Figure 8:**
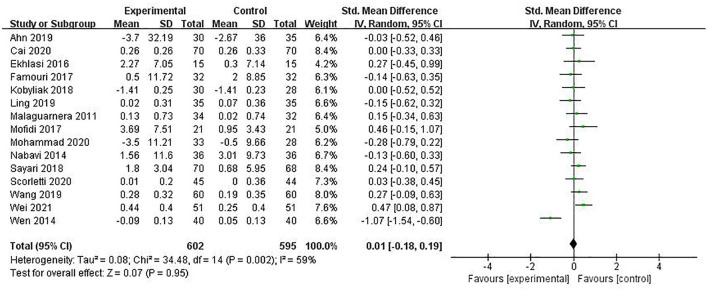
The role of PPS in LDL-C in patients with NAFLD.

HDL-C was reported in 15 studies involving 1,197 patients, and the pooled estimate was SMD = 0.01, (95% CI [−0.18, 0.19], *P* = 0.95, *I*^2^ = 59%, random-effects model), with no statistically significant difference (see Figure 8).

#### LDL-C

**Figure 9:**
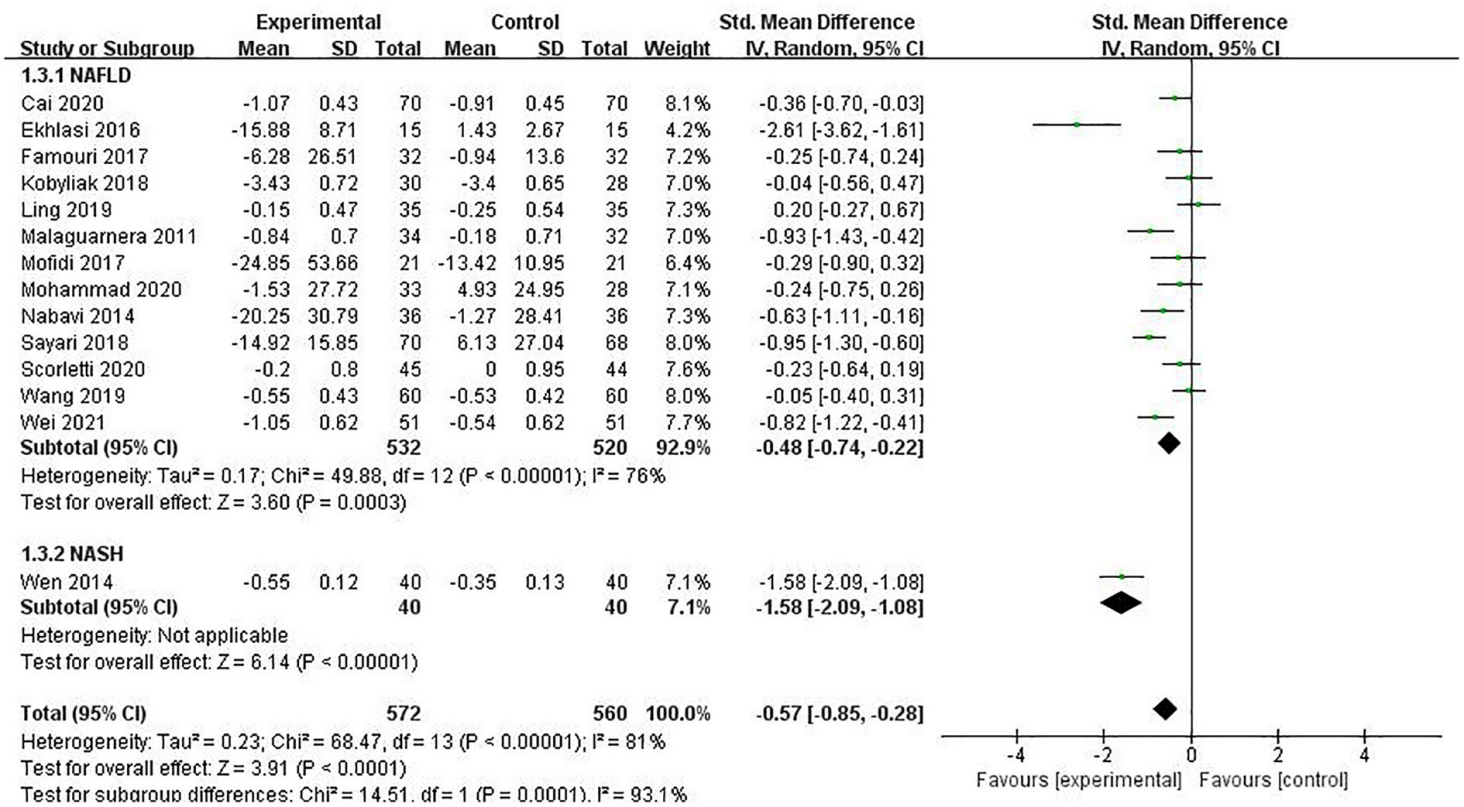
The role of PPS in HDL-C in patients with NAFLD.

LDL-C was reported in 14 studies involving 1,132 patients, and the combined estimate was SMD = −0.57 (95% CI [−0.85, −0.28], *P* < 0.00001, *I*^2^ = 81%), with a statistically significant difference. Subgroup analysis of the included studies revealed a statistically significant difference between the two groups according to disease type (*P* = 0.0001), but the reliability of inter-group differences was insufficient as only one study was included in the NASH group. Overall meta-analysis results suggested that PPS regulation could reduce LDL-C in NAFLD patients, as shown in Figure 9.

### Effect on protein metabolism

A meta-analysis was carried out on the protein metabolism indicator of ALB. And we also conducted subgroup analyses, based primarily on the types of intervention (probiotic, prebiotic, synbiotic), type of control (placebo/non placebo), but no significant difference was found between subgroups (see in Supplementary Figures S28, S29).

#### ALB

**Figure 10:**
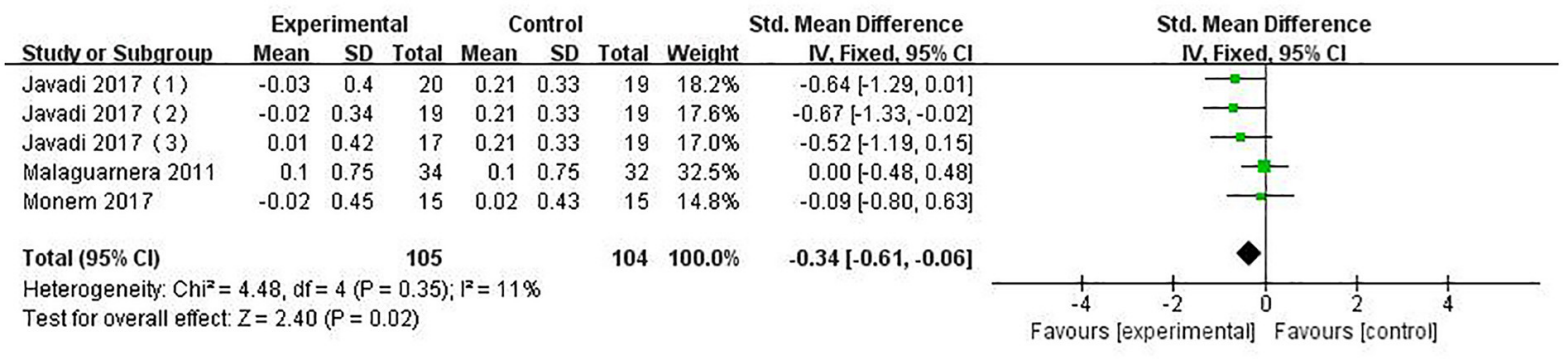
The role of PPS in ALB in patients with NAFLD.

The indicator of ALB was reported in five studies involving 209 patients. The combined estimated value was SMD = −0.34 (95%CI [−0.61, −0.06], *P* = 0.02, *I*^2^ = 11%, fixed-effects model), and the difference was statistically significant, suggesting that PPS regulation could reduce ALB in NAFLD patients (see Figure 10).

### Sensitivity analysis and publication bias

Meta-analysis and sensitivity analysis was conducted on all preset indicators, and sensitivity analysis showed no changes in robustness except for the indicator of HOMA-IR. The funnel plots of glucose, HOMA-IR, TC, TG, LDL-C, and HDL-C were analyzed respectively, publication bias was qualified by Egger's test and found in HOMA-IR, TG, LDL-C and HDL-C, no significant differences were found after utilizing trim-and-fill method (see in Supplementary Figures S30–S35).

## Discussion

The current study systematically evaluated the effects of PPS regulation on energy metabolism biomarkers in NAFLD patients. The results showed that PPS regulation significantly improved the sugar and lipid metabolism indicators of NAFLD, and may have a negative impact on protein metabolism. Subgroup analysis, based on the types of disease (NAFLD/NASH), types of intervention(probiotic, prebiotic, synbiotic), type of control(placebo/non placebo), administrating duration( ≤ 12 weeks; >12 weeks, <24 weeks; ≥24 weeks), shows no statistically difference. No significant publication bias was identified.

According to the results of this study, the PPS regulation method may reduce the levels of glucose, HOMA-IR and insulin in NAFLD patients, and thus play a certain role in regulating the sugar metabolism level of patients. In 70–80% of NAFLD patients, there is a problem of elevated blood glucose (3), and studies by Mirmiranpour et al. have shown that PPS regulation can reduce blood glucose levels (49). PPS may improve the blood glucose problem in NAFLD by changing intestinal microflora and stimulating the production of glucose-dependent insulinotropic polypeptide (GIP) and glucagon-like peptides (GLPs), thereby increasing glucose uptake and lowering blood glucose levels (50). This study showed that PPS regulation reduced the glucose level in NAFLD (SMD = −0.23, 95%CI [−0.45, −0.01], *P* = 0.04), which is consistent with the above research. Insulin resistance is one of the characteristics of NAFLD (51), and HOMA-IR is the most widely used model method to evaluate insulin resistance in microbial therapy. The study by Nazarii et al. showed that PPS regulation can reduce the HOMA-IR level and improve insulin resistance (52). Previous studies have suggested that PPS may increase insulin sensitivity and improve NAFLD insulin resistance by regulating the NF-κB signaling pathway, reducing the expression of TNF-α and inflammatory response (53). Insulin resistance is associated with increased LPS levels, and impaired intestinal barrier leads to increased LPS levels in the circulation (54). PPS regulation may improve insulin resistance by regulating the production of tight junction protein mucin, improving the non-specific intestinal barrier defense mechanism, and reducing LPS levels (17, 55). The results of this study showed that PPS regulation could reduce the level of HOMA-IR (SMD = −0.47, 95%CI [−0.63, −0.31], *P* < 0.00001), suggesting that PPS regulation might improve insulin resistance in NAFLD. Elevated serum fasting insulin level is regarded as one of the main pathogenic factors of NAFLD (56), and PPS regulation is considered to reduce insulin levels (57). The exact mechanism by which the PPS regulation method affects insulin levels is unclear, and since PPS use glucose as the primary energy source, their effects on serum insulin levels may be mediated by affecting blood glucose levels (58). The results of this study showed that PPS regulation could reduce insulin levels (SMD = −0.46, 95% CI [−0.76, −0.16], *P* = 0.002), further providing evidence-based proof for this view.

This study showed that PPS regulation could reduce the levels of TC and LDL-C in NAFLD patients, while there was no significant difference in the indicators of TG and HDL-C. Elevated TC is an important risk factor for the pathogenesis of NAFLD (59), and low HDL-C and high LDL-C are characteristics of NAFLD (60, 61). Studies have shown that PPS regulation can reduce TC and LDL-C levels and increase HDL-C levels (62), and PPS may regulate cholesterol metabolism in NAFLD patients. The results of this study showed that PPS regulation could reduce the levels of TC (SMD = −0.62, 95%CI [−0.87, −0.36], *P* < 0.00001) and LDL-C (SMD = −0.57, 95%CI [−0.85, −0.28], *P* < 0.00001). Although there was no statistical difference in HDL-C, the overall effect size suggested that PPS regulation could increase the HDL-C level [SMD = 0.01, (95%CI [−0.18, 0.19], *P* = 0.95], providing support for the view that microbial therapy can regulate cholesterol metabolism. Elevated TG is also a risk factor for NAFLD (59), and studies have shown that PPS regulation can reduce the TG level (62). PPS may change the gene expression of lipogenic enzymes and reduce the *de novo* synthesis of fatty acids in the liver, thereby reducing the accumulation of triglycerides in the liver (63); TG levels are reduced by inhibiting the transcription of carbohydrate response element binding protein (ChREBP) and activating the transcription of peroxisome proliferator-activated receptor alpha (PPARα) encoding genes (64). Although the analysis results of this study showed no statistically significant difference in the effect of PPS regulation on the indicator of TG, the overall effect size suggested that the TG level decreased (SMD = −0.24, 95%CI [−0.59, −0.12], *P* = 0.19), indicating that PPS regulation may reduce the TG level in NAFLD.

This study also found that PPS regulation may reduce ALB levels (SMD = −0.34, 95% CI [−0.61, −0.06], *P* = 0.02). Intestinal microbiota can produce amino acids by fermenting dietary proteins, utilize amino acids for protein synthesis, and catabolize amino acids through deamination and decarboxylation (65–67). As an important protein in the human body, ALB plays an important role in maintaining body nutrition and osmotic pressure, and is the most important indicator reflecting liver synthesis function (68). A study showed that ALB level was negatively correlated with liver fibrosis indicators of LN and PC III in NAFLD patients (69). There are few previous studies on the effect of PPS regulation on protein metabolism. Considering the important role of intestinal microbiota in the process of protein synthesis and metabolism and the vital regulatory effect of microbial therapy on intestinal microbiota, PPS regulation may affect protein metabolism of NAFLD by regulating intestinal microbiota. However, this study only included five groups of comparisons on the indicator of ALB, three of which were from the same study. Due to limited data sources, the results should be interpreted with caution.

NASH is a progressive form of NAFLD. This study showed that PPS regulation could reduce the levels of Glucose, Insulin, TC, TG, HDL-C and LDL-C in NASH patients. Studies have shown that PPS regulation could prevent obesity and improve liver histology in NASH, which might be outcomes of regulating aforementioned indicators (16, 70). Moreover, in animal models, PPS could accelerate the lowering of plasma glucose levels during an insulin tolerance test in diet-induced obesity mice, and plays a certain role in regulating the sugar metabolism (71). Bile acid (BA) is essential for lipid and carbohydrate metabolism (72). PPS has been found possessing a certain effect on regulating BA synthesis to control lipid and sugar metabolism, and furthermore resisting the development of NASH (73). Despite aforementioned evidences, we have not found more evidences to explore the differences in the effects of PPS on NAFLD and NASH in terms of sugar, lipids, and proteins metabolism, future research might be needed.

On the basis of what we found, administration of PPS might be applied in more clinical scenario. Supplementation of PPS to regulate the gut microbiota should be explorably applied in the conventional treatment of NAFLD for PPS might has the potential to prevent the progression of NAFLD to NASH. PPS could reduce glucose, insulin and insulin resistance levels in NAFLD patients by restoring the homeostasis of gut microbiota, improved glucose metabolism disorders, and has application advantages in diseases including NAFLD and type 2 diabetes (T2D) which generally comorbid with abnormal glucose metabolism (26, 38). PPS could improve lipid metabolism disorder and reduced blood lipid and cholesterol levels in NAFLD patients. The potential mechanisms related to the hypocholesterolemic effect was that PPS could prevent free bile acids reabsorption and compensatory increased use of cholesterol to produce bile acids, which could lead to a reduction in the cholesterol present in serum (74). Evidence supports that PPS can improve cardiovascular disease risk factors, and it is suggested that it could be considered in the treatment of atherosclerosis and hypertension (75–77). This study showed that microbial therapy may reduce the ALB level of NAFLD patients, suggesting that we should pay attention to the impact of microbial therapy on protein metabolism and timely supplement protein when treating NAFLD.

The present study also has some limitations. (1) The sample size of the included studies was small with few relevant studies on certain indicators, and there were differences in the types, doses, intervention time of probiotics and lifestyle management in the included RCTs. In addition, a few of the trial designs were not standardized enough, which affected the effectiveness of the evaluation. (2) The included study involved adverse reactions of microbial therapy, and its side effects on NAFLD need to be further studied. (3) Although the risk of bias result for this study was medium and low, we couldn't be sure what impact of the risk would be. (4) Because the inconsistent bacteria strains and dose, a dose-response analysis was not applicable, more attention should be paid to this problem in further studies.

## Conclusions

The results of this study suggest that microbial therapy has a certain effect on the energy metabolism of NAFLD, and can be used as a new treatment option for NAFLD, but meanwhile, attention should be paid to its effect on protein reduction. However, the results of our study should be interpretated cautiously for the limitations in our study.

## Data availability statement

The original contributions presented in the study are included in the article/supplementary material, further inquiries can be directed to the corresponding author/s.

## Author contributions

Conceptualization: ShL and SuL. Methodology: ZW, FD, ZJ, and XC. Software: ShL, ZW, and JL. Validation, writing—review and editing, and supervision: ShL, SuL, JL, FD, ZJ, XC, and ZW. Writing—original draft preparation: ShL. Funding acquisition: SuL, XC, and FD. All authors have read, critically appraised, and agreed to the published version of the manuscript.

## Funding

This study was funded by the National Science and Technology major projects (Project Code: 2018ZX10303502); China Postdoctoral Science Foundation Special Funding for New Coronary Pneumonia Epidemic Prevention and Control (Project Code: 2020T130032ZX); Scientific research project of Henan Provincial Administration of Traditional Chinese Medicine (No. 20-21ZY1011; No. 2022ZY1002; No. 2018ZY2033).

## Conflict of interest

The authors declare that the research was conducted in the absence of any commercial or financial relationships that could be construed as a potential conflict of interest.

## Publisher's note

All claims expressed in this article are solely those of the authors and do not necessarily represent those of their affiliated organizations, or those of the publisher, the editors and the reviewers. Any product that may be evaluated in this article, or claim that may be made by its manufacturer, is not guaranteed or endorsed by the publisher.
