# Supplementary material for: The promising role of probiotics/prebiotics/synbiotics in energy metabolism biomarkers in patients with NAFLD: A systematic review and meta-analysis
Source: Front Public Health. 2022 Jul 25;10:862266. doi: 10.3389/fpubh.2022.862266 (PMC9358257; doi:10.3389/fpubh.2022.862266)
Supplement: Supplementary file 1 [file Data_Sheet_1.docx]

Table S1: Search strategy

| database | # | Query | Results |
| --- | --- | --- | --- |
| Pubmed | 13  12  11  10  9  8  7  6  5  4  3  2  1 | #11 AND #12  (#2 OR #7) OR (#3 OR #8) OR (#4 OR #9) OR (#5 OR #10)  #1 OR #6  (((((((((((((dietary Fibers[Title/Abstract]) OR (fibers, Dietary[Title/Abstract])) OR (fiber, Dietary[Title/Abstract])) OR (wheat Bran[Title/Abstract])) OR (bran, Wheat[Title/Abstract])) OR (brans, Wheat[Title/Abstract])) OR (wheat Brans[Title/Abstract])) OR (roughage[Title/Abstract])) OR (roughages[Title/Abstract])) OR (alimentary fiber[Title/Abstract])) OR (diet fiber[Title/Abstract])) OR (dietary fibre[Title/Abstract])) OR (optifibre[Title/Abstract])) OR (stimulance multi fibre[Title/Abstract])  ((synbiotic agent[Title/Abstract]) OR (synbiotic[Title/Abstract])) OR (synbiotics[Title/Abstract])  ((prebiotic agent[Title/Abstract]) OR (prebiotic[Title/Abstract])) OR (prebiotics[Title/Abstract])  ((probiotic agent[Title/Abstract]) OR (probiotic[Title/Abstract])) OR (probiotics[Title/Abstract])  (((((((((((((((((((((((((Non alcoholic Fatty Liver Disease[Title/Abstract]) OR (NAFLD[Title/Abstract])) OR (Nonalcoholic Fatty Liver Disease[Title/Abstract])) OR (Fatty Liver, Nonalcoholic[Title/Abstract])) OR (Fatty Livers, Nonalcoholic[Title/Abstract])) OR (Liver, Nonalcoholic Fatty[Title/Abstract])) OR (Livers, Nonalcoholic Fatty[Title/Abstract])) OR (Nonalcoholic Fatty Liver[Title/Abstract])) OR (Nonalcoholic Fatty Livers[Title/Abstract])) OR (Nonalcoholic Steatohepatitis[Title/Abstract])) OR (Nonalcoholic Steatohepatitides[Title/Abstract])) OR (Steatohepatitides, Nonalcoholic[Title/Abstract])) OR (Steatohepatitis, Nonalcoholic[Title/Abstract])) OR (non alcoholic fatty liver disease[Title/Abstract])) OR (non alcoholic hepato-steatosis[Title/Abstract])) OR (non alcoholic hepatosteatosis[Title/Abstract])) OR (non alcoholic liver steatosis[Title/Abstract])) OR (non alcoholic steatotic hepatopathy[Title/Abstract])) OR (non-alcoholic fatty liver[Title/Abstract])) OR (non-alcoholic fatty liver disease[Title/Abstract])) OR (non-alcoholic FLD[Title/Abstract])) OR (non-alcoholic hepatic steatosis[Title/Abstract])) OR (nonalcoholic FLD[Title/Abstract])) OR (nonalcoholic hepatic steatosis[Title/Abstract])) OR (nonalcoholic hepatosteatosis[Title/Abstract])) OR (nonalcoholic liver steatosis[Title/Abstract])  "Dietary Fiber"[Mesh]  "Synbiotics"[Mesh]  "Prebiotics"[Mesh]  "Probiotics"[Mesh]  "Non-alcoholic Fatty Liver Disease"[Mesh] | 516  62,727  31,624  9,000  1,829  10,378  30,088  30,186  21,499  791  3,388  20,355  16,523 |
| Embase | 17  16  15  14  13  12  11  10  9  8  7  6  5  4  3  2  1 | #15 NOT #16  'rat':ti OR 'rats':ti OR 'mouse':ti OR 'mice':ti OR 'dog':ti OR 'pig':ti  #13 NOT #14  'review':it OR 'letter':it OR 'comment':it OR 'editorial':it  #11 AND #12  #2 OR #7 OR #3 OR #8 OR #4 OR #9 OR #5 OR #10  #1 OR #6  'dietary fibers':ti,ab,kw OR 'fibers, dietary':ti,ab,kw OR 'fiber, dietary':ti,ab,kw OR 'wheat bran':ti,ab,kw OR 'bran, wheat':ti,ab,kw OR 'brans, wheat':ti,ab,kw OR 'wheat brans':ti,ab,kw OR 'roughage':ti,ab,kw OR 'roughages':ti,ab,kw OR 'alimentary fiber':ti,ab,kw OR 'diet fiber':ti,ab,kw OR 'dietary fibre':ti,ab,kw OR 'optifibre':ti,ab,kw OR 'stimulance multi fibre':ti,ab,kw  'synbiotic agent':ti,ab,kw OR 'synbiotic':ti,ab,kw OR 'synbiotics':ti,ab,kw  'prebiotic agent':ti,ab,kw OR 'prebiotic':ti,ab,kw OR 'prebiotics':ti,ab,kw  'probiotic agent':ti,ab,kw OR 'probiotic':ti,ab,kw OR 'probiotics':ti,ab,kw  'nafld':ti,ab,kw OR 'nonalcoholic fatty liver disease':ti,ab,kw OR 'fatty liver, nonalcoholic':ti,ab,kw OR 'fatty livers, nonalcoholic':ti,ab,kw OR 'liver, nonalcoholic fatty':ti,ab,kw OR 'livers, nonalcoholic fatty':ti,ab,kw OR 'nonalcoholic fatty liver':ti,ab,kw OR 'nonalcoholic fatty livers':ti,ab,kw OR 'nonalcoholic steatohepatitis':ti,ab,kw OR 'nonalcoholic steatohepatitides':ti,ab,kw OR 'steatohepatitides, nonalcoholic':ti,ab,kw OR 'steatohepatitis, nonalcoholic':ti,ab,kw OR 'non alcoholic fatty liver disease':ti,ab,kw OR 'non alcoholic hepato-steatosis':ti,ab,kw OR 'non alcoholic hepatosteatosis':ti,ab,kw OR 'non alcoholic liver steatosis':ti,ab,kw OR 'non alcoholic steatotic hepatopathy':ti,ab,kw OR 'non-alcoholic fatty liver':ti,ab,kw OR 'non-alcoholic fatty liver disease':ti,ab,kw OR 'non-alcoholic fld':ti,ab,kw OR 'non-alcoholic hepatic steatosis':ti,ab,kw OR 'nonalcoholic fld':ti,ab,kw OR 'nonalcoholic hepatic steatosis':ti,ab,kw OR 'nonalcoholic hepatosteatosis':ti,ab,kw OR 'nonalcoholic liver steatosis':ti,ab,kw  'dietary fiber'/exp  'synbiotic agent'/exp  'prebiotic agent'/exp  'probiotic agent'/exp  'nonalcoholic fatty liver'/exp | 520  1776046  672  4721198  1228  86650  60181  11020  2291  12405  38676  47631  32966  2206  9656  42636  52749 |
| Cochrane Library | 1  2  3  4  5  6  7  8  9  10  11  12  13 | MeSH descriptor: [Non-alcoholic Fatty Liver Disease] explode all trees  (“Non alcoholic Fatty Liver Disease” OR “NAFLD” OR “Nonalcoholic Fatty Liver Disease” OR “Fatty Liver, Nonalcoholic” OR “Fatty Livers, Nonalcoholic” OR “Liver, Nonalcoholic Fatty” OR “Livers, Nonalcoholic Fatty” OR “Nonalcoholic Fatty Liver” OR “Nonalcoholic Fatty Livers” OR “Nonalcoholic Steatohepatitis” OR “Nonalcoholic Steatohepatitides” OR “Steatohepatitides, Nonalcoholic” OR “Steatohepatitis, Nonalcoholic” OR “non alcoholic fatty liver disease” OR “non alcoholic hepato-steatosis” OR “non alcoholic hepatosteatosis” OR “non alcoholic liver steatosis” OR “non alcoholic steatotic hepatopathy” OR “non-alcoholic fatty liver” OR “non-alcoholic fatty liver disease” OR “non-alcoholic FLD” OR “non-alcoholic hepatic steatosis” OR “nonalcoholic FLD” OR “nonalcoholic hepatic steatosis” OR “nonalcoholic hepatosteatosis” OR “nonalcoholic liver steatosis”):ti,ab,kw  MeSH descriptor: [Probiotics] explode all trees  (“probiotic agent” OR “probiotic” OR “probiotics”):ti,ab,kw  MeSH descriptor: [Prebiotics] explode all trees  (“prebiotic agent” OR “prebiotic” OR “prebiotics”):ti,ab,kw  MeSH descriptor: [Synbiotics] explode all trees  (“synbiotic agent” OR “synbiotic” OR “synbiotics”):ti,ab,kw  MeSH descriptor: [Dietary Fiber] explode all trees  (“dietary Fibers” OR “fibers, Dietary” OR “fiber, Dietary” OR “wheat Bran” OR “bran, Wheat” OR “brans, Wheat” OR “wheat Brans” OR “roughage” OR “roughages” OR “alimentary fiber” OR “diet fiber” OR “dietary fibre” OR “optifibre” OR “stimulance multi fibre”):ti,ab,kw  #1 OR #2  (#3 OR #4) OR (#5 OR #6) OR (#7 OR #8) OR (#9 OR #10)  #11 AND #12 | 1143  3678  2267  7747  321  1726  177  839  2105  3678  3678  12490  177 |
| CNKI | - | （主题：非酒精性脂肪性肝病（精确））OR（主题：NAFLD（精确））OR （主题：非酒精性脂肪肝（精确））OR （主题：非酒精性脂肪肝病（精确））OR （主题：单纯性脂肪肝（精确））AND（（主题：益生菌（精确））OR（主题：益生元（精确））OR（主题：合生元（精确））OR（主题：膳食纤维（精确））） | 202 |
| CT.gov | - | 1 Study found for: probiotics \| Studies With Results \| Non-alcoholic Fatty Liver Disease  Applied Filters: With Results  Investigation of Synbiotic Treatment in NAFLD  1 Study found for: prebiotics \| Studies With Results \| Non-alcoholic Fatty Liver Disease  Applied Filters: With Results  Investigation of Synbiotic Treatment in NAFLD  1 Study found for: synbiotics \| Studies With Results \| Non-alcoholic Fatty Liver Disease  Applied Filters: With Results  Investigation of Synbiotic Treatment in NAFLD  1 Study found for: dietary fiber \| Studies With Results \| Non-alcoholic Fatty Liver Disease  Applied Filters: With Results  Fucoidan Improves the Metabolic Profiles of Patients With Non-alcoholic Fatty Liver Disease (NAFLD) | 2 |

**
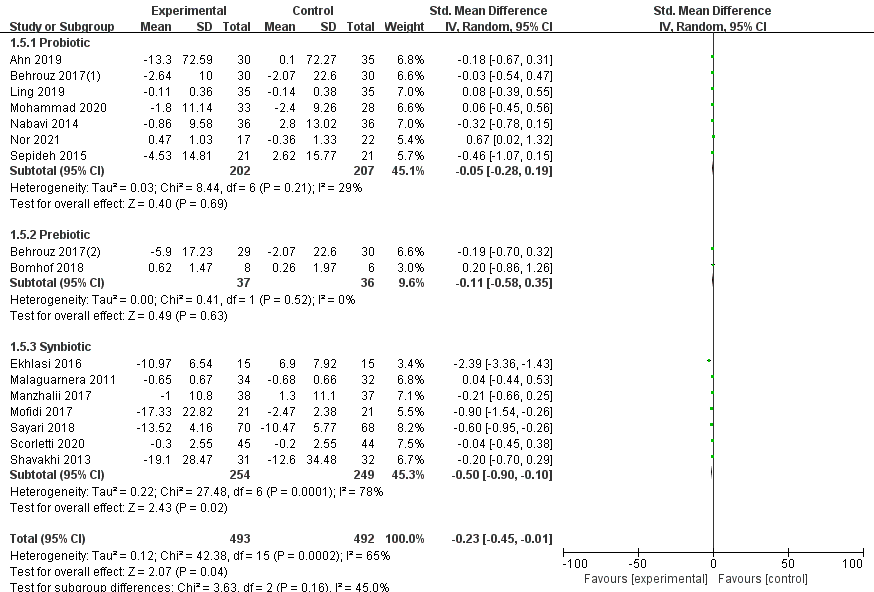

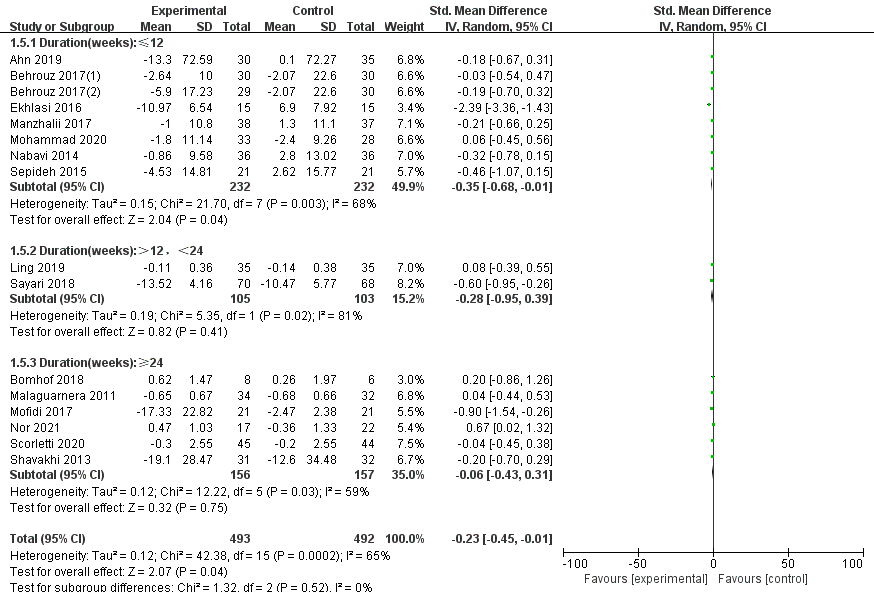
**

Figure S1. Glucose- type of intervention Figure S2. Glucose-duration

**
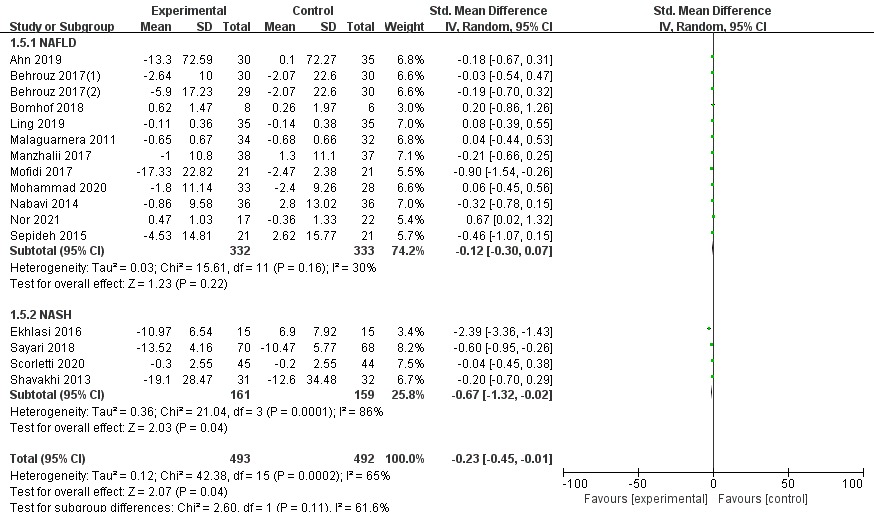

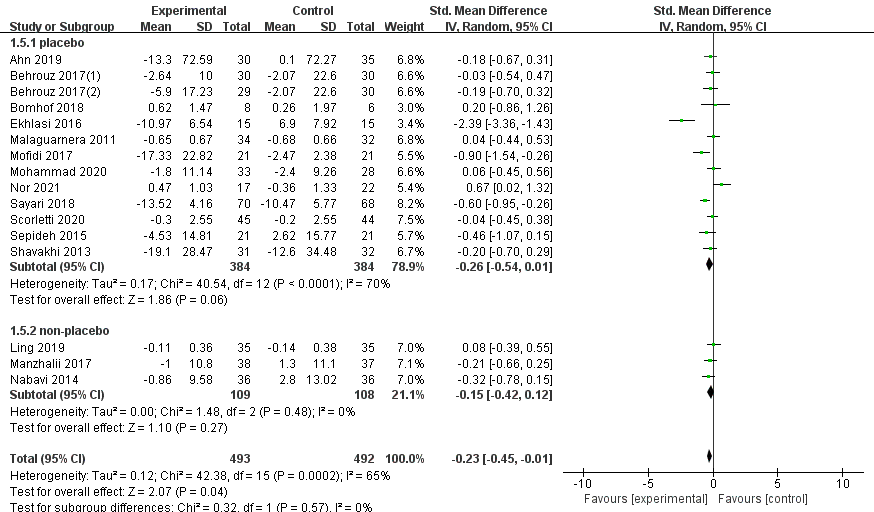
**

Figure S3. Glucose- type of disease Figure S4. Glucose- type of control

**
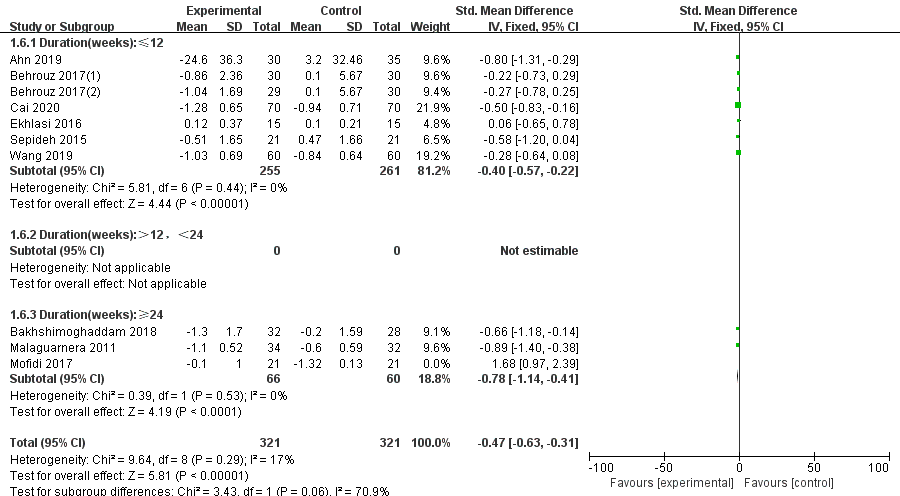

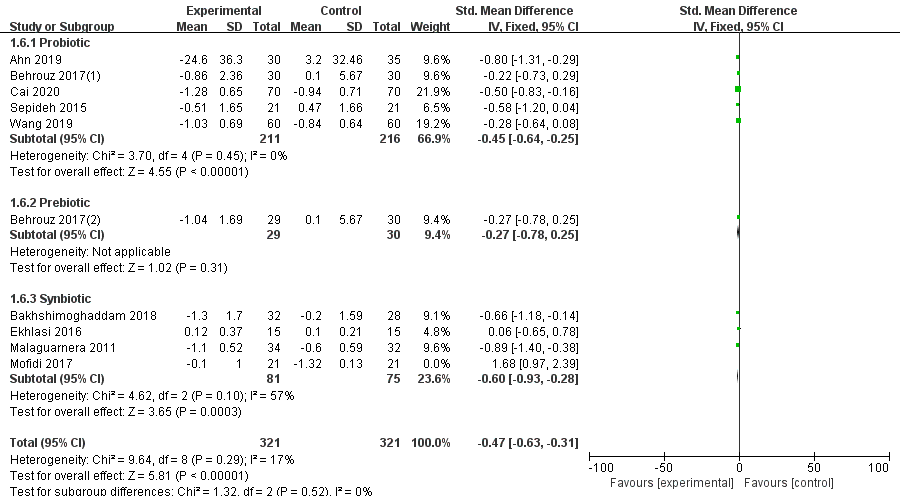
**

Figure S5. HOMA-IR- duration Figure S6.HOMA-IR-type of intervention

**
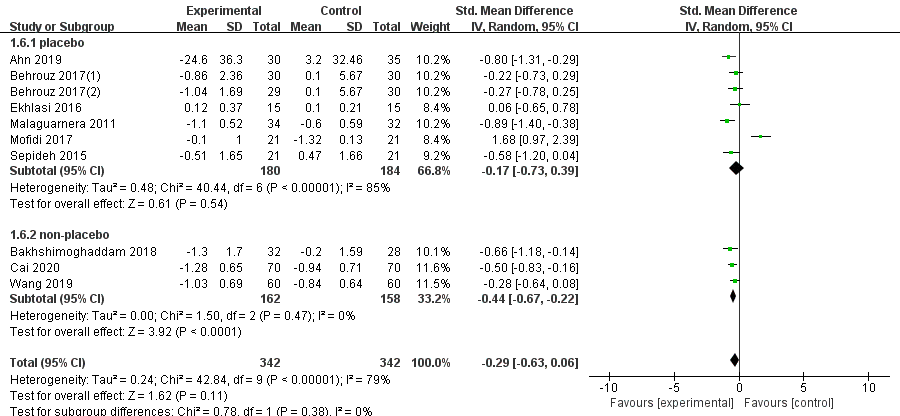

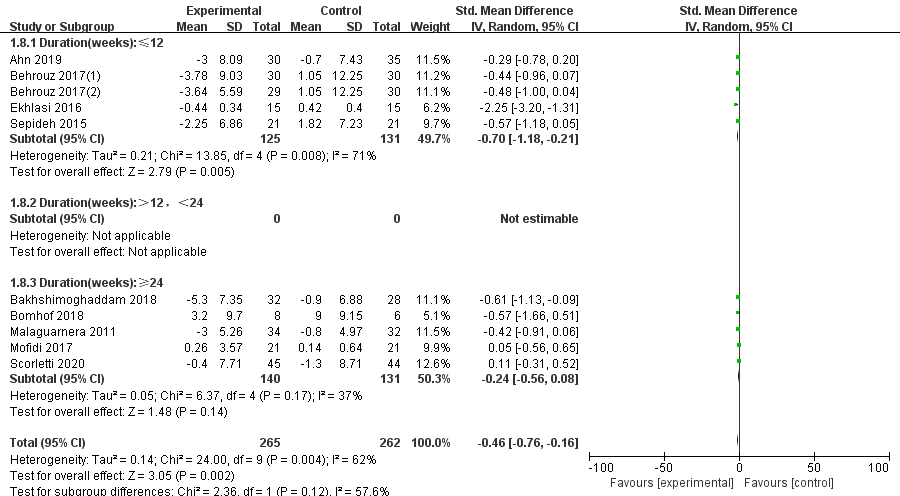
**

Figure S7.HOMA-IR-type of control Figure S8.Insulin-duration

**
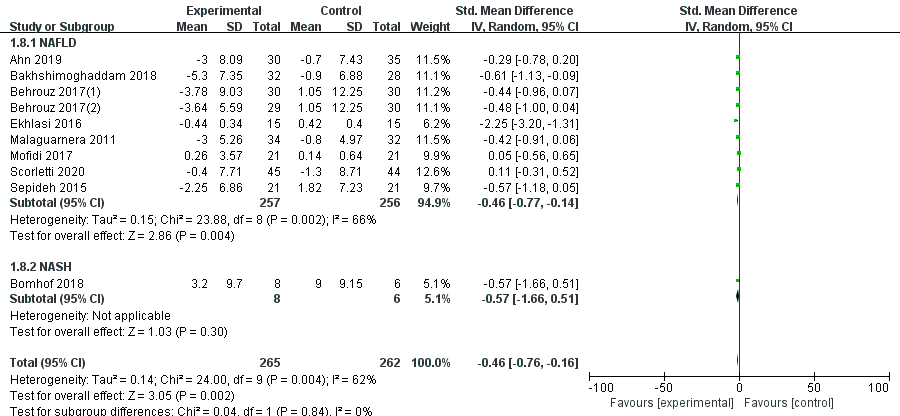

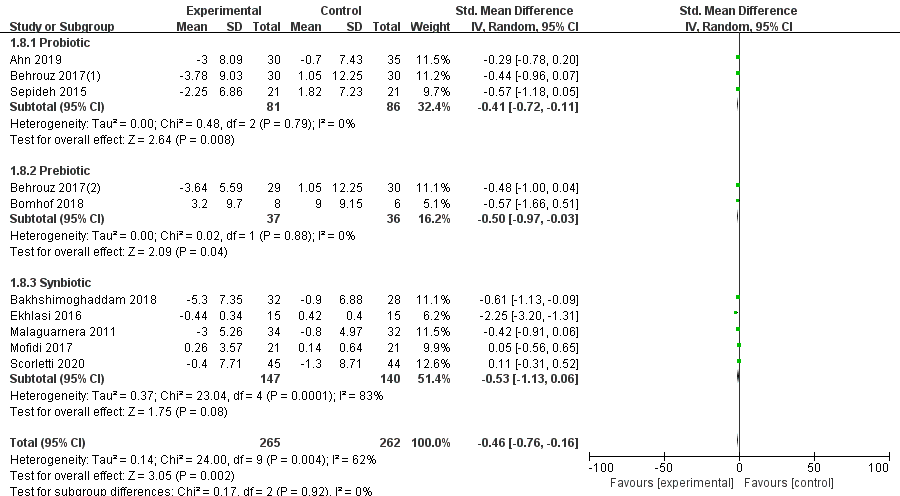
**

Figure S9.Insulin-type of disease Figure S10.Insulin-type of intervention

**
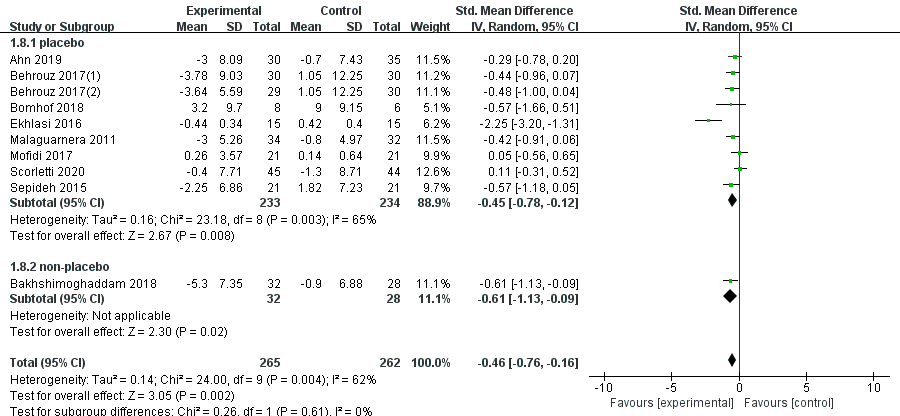

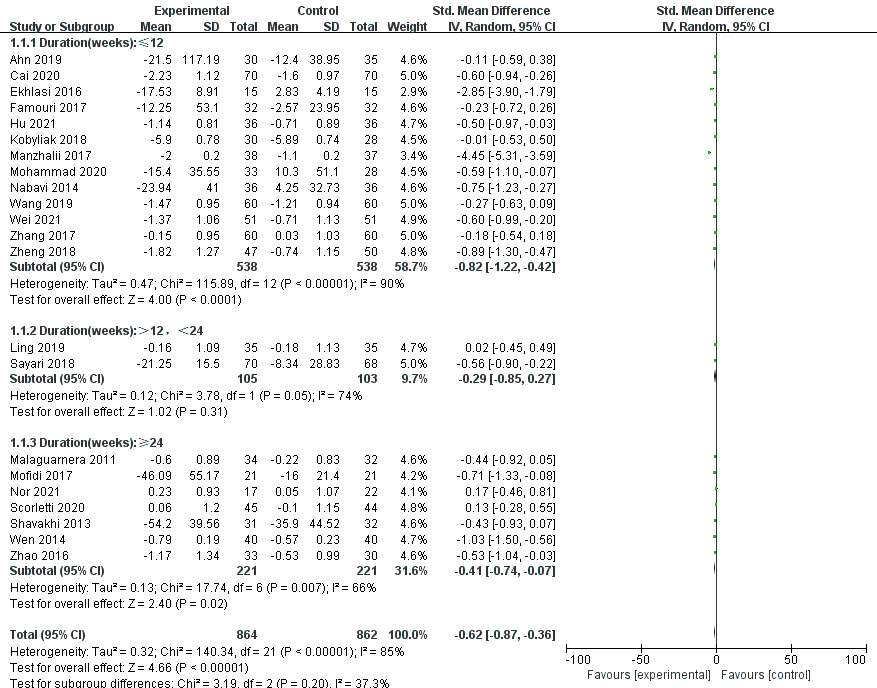
**

Figure S11.Insulin-type of control Figure S12.TC-duration

**
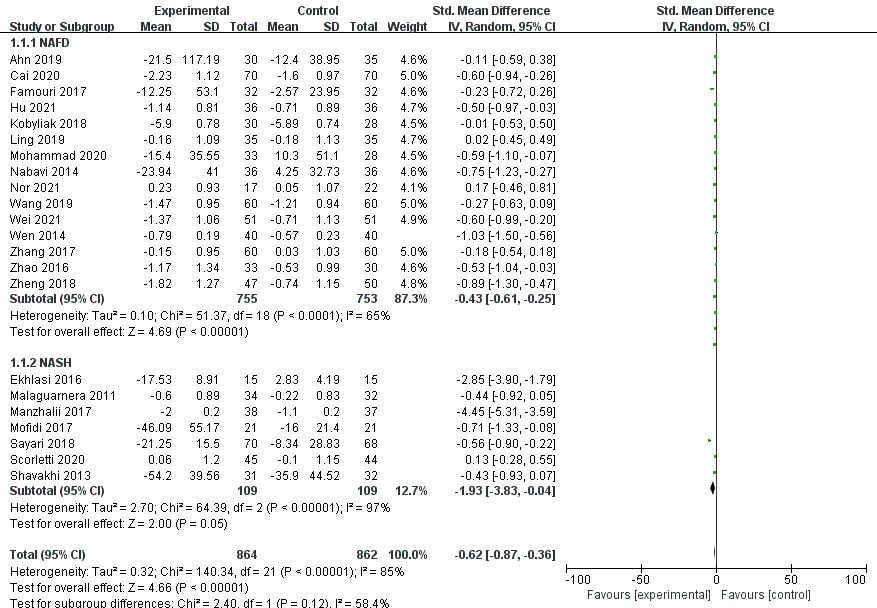

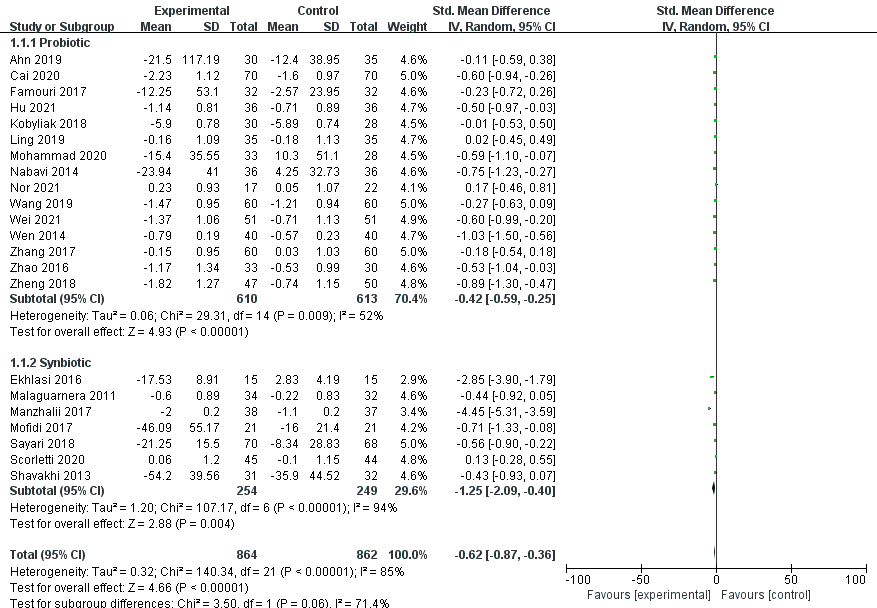
**

Figure S13.TC-type of disease Figure S14.TC-type of intervention

**
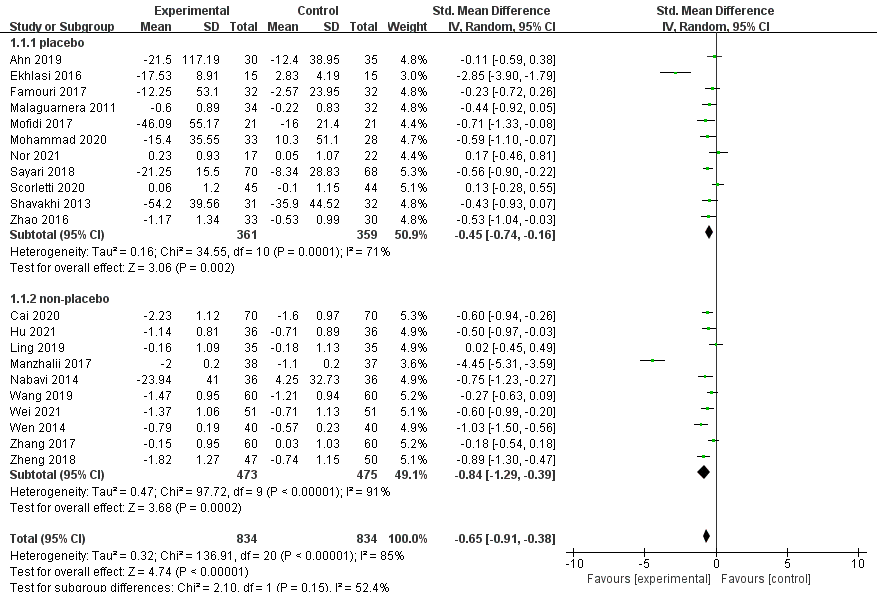

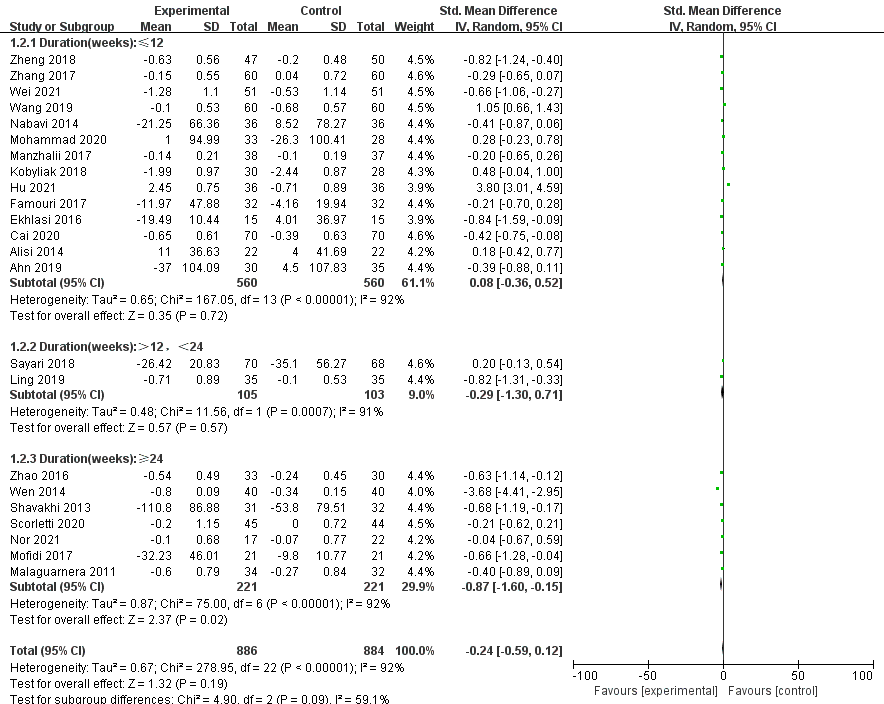
**

Figure S15.TC-type of control Figure S16.TG-duration

**
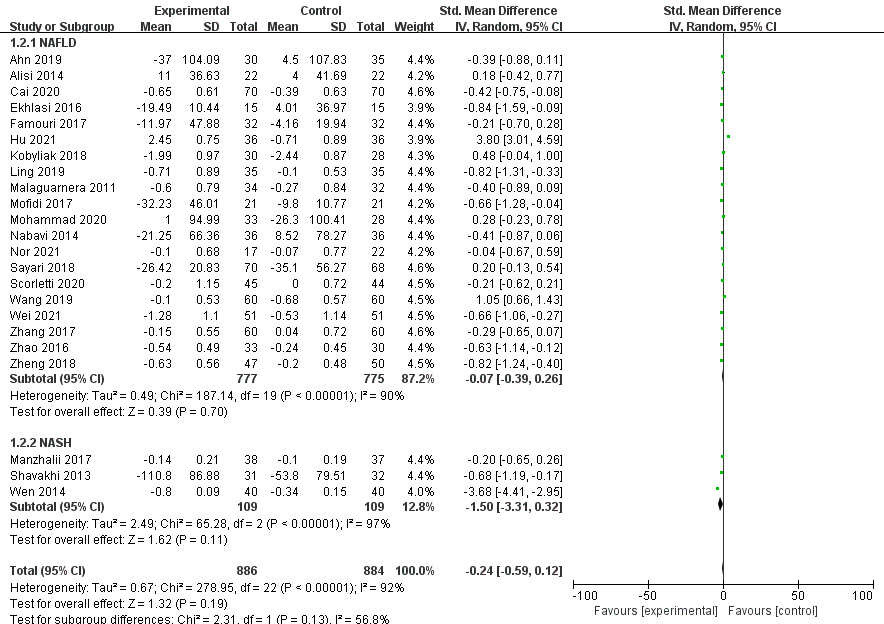

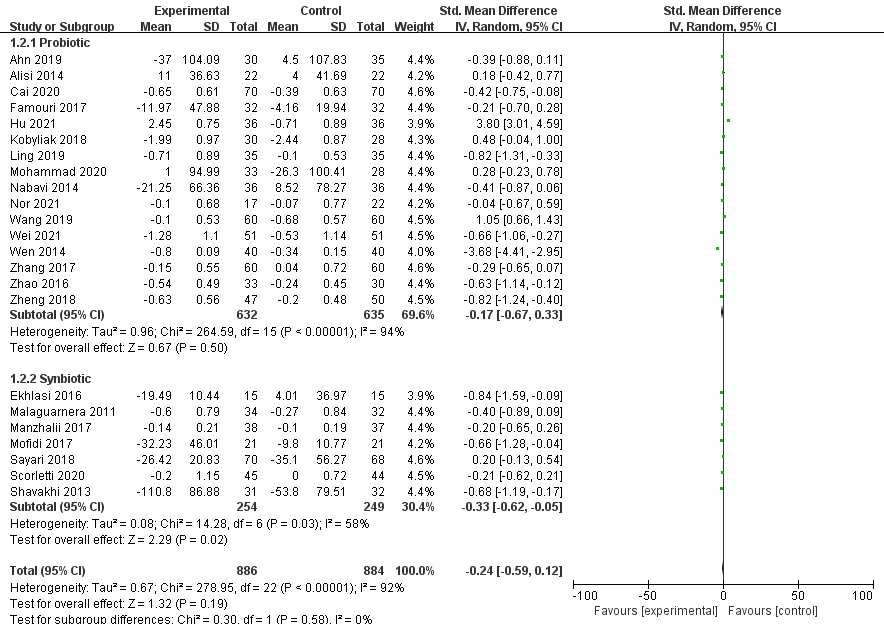
**

Figure S17.TG-type of disease Figure S18.TG-type of intervention

**
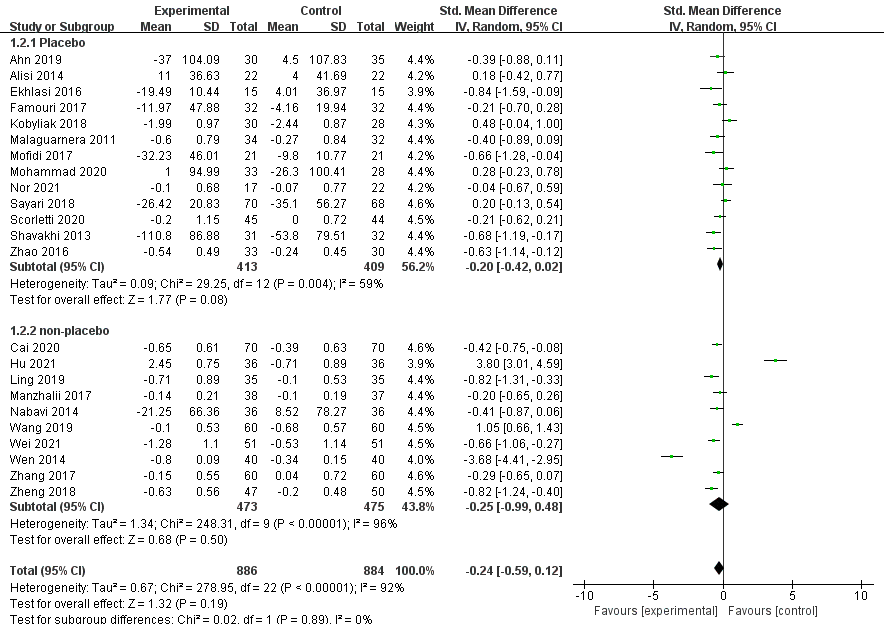

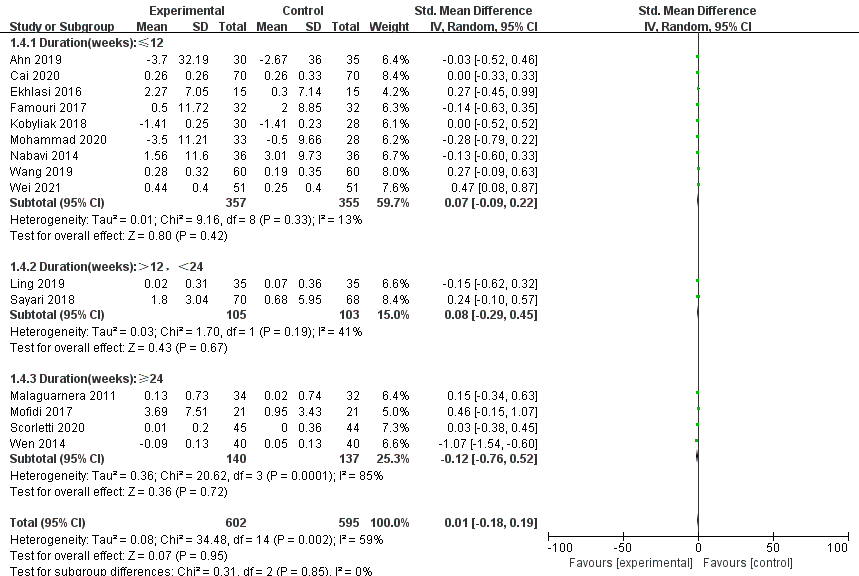
**

Figure S19.TG-type of control Figure S20.HDL-C-duration

**
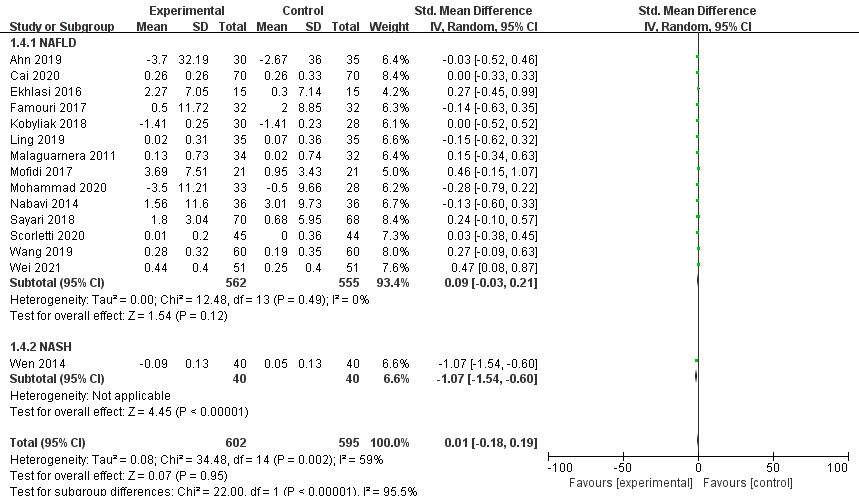

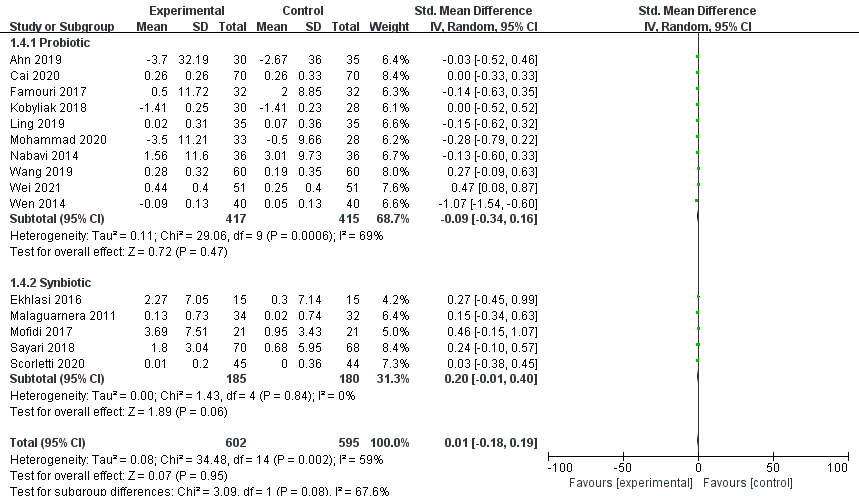
**

Figure S21.HDL-C-type of disease Figure S22.HDL-C-type of intervention

**
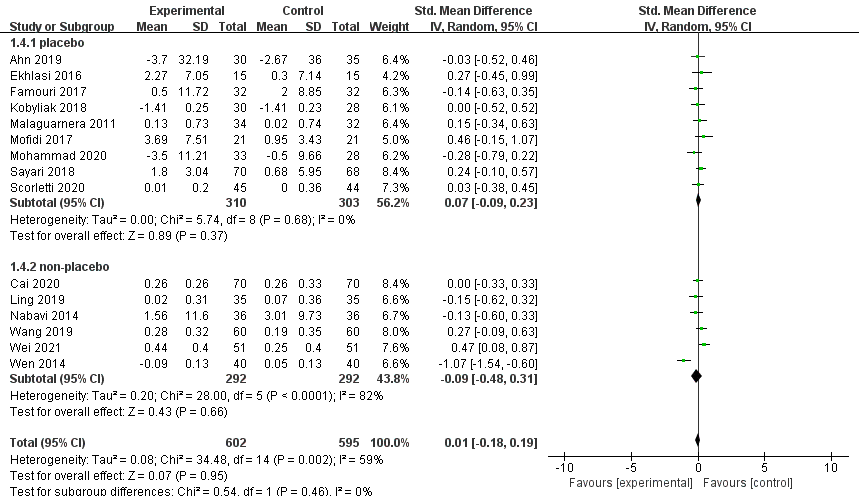

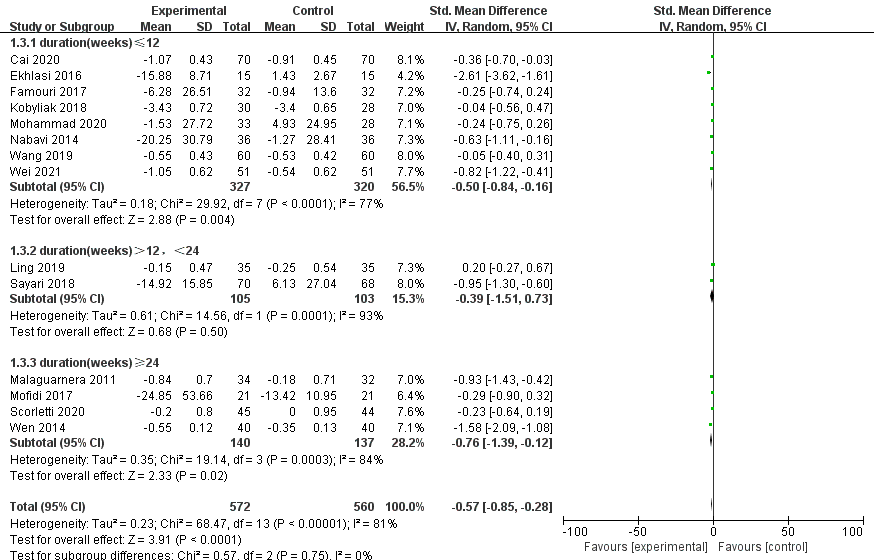
**

Figure S23.HDL-C-type of control Figure S24.LDL-C-duration

**
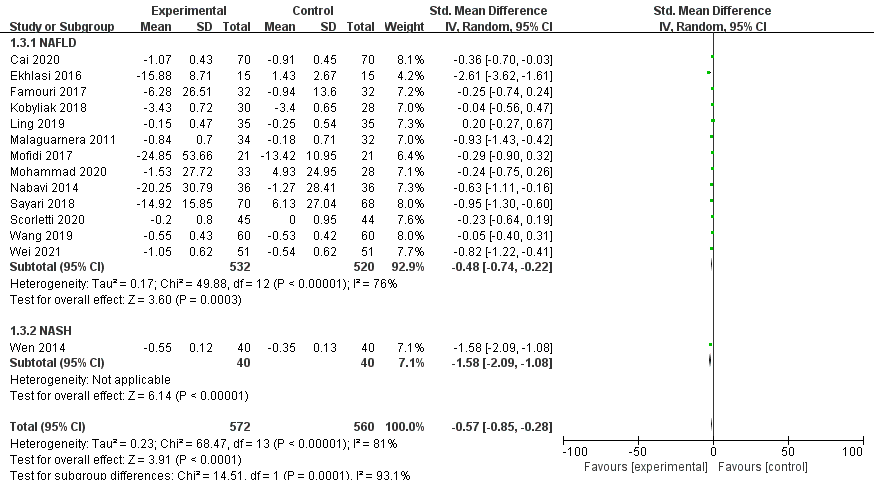

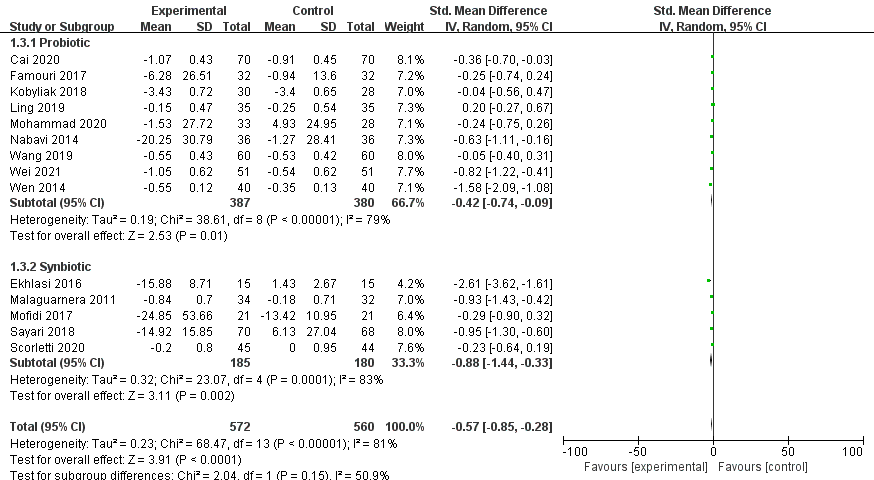
**

Figure S25.LDL-C-type of disease Figure S26.LDL-C-type of intervention


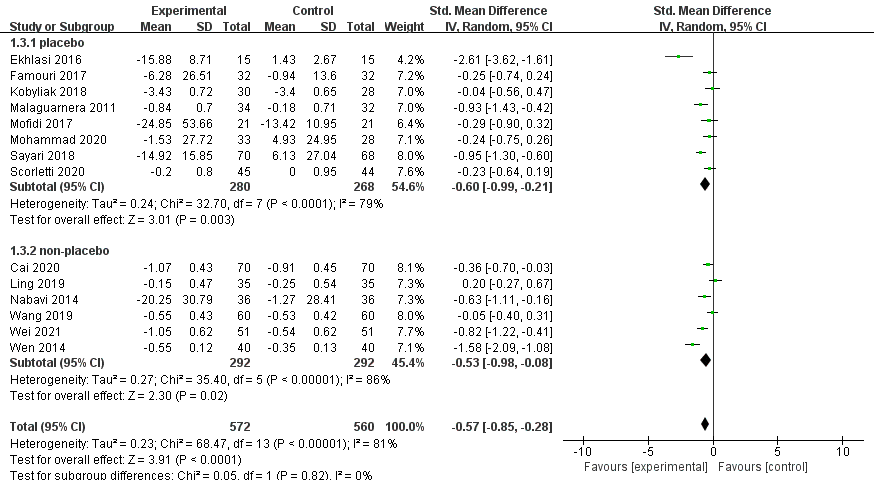

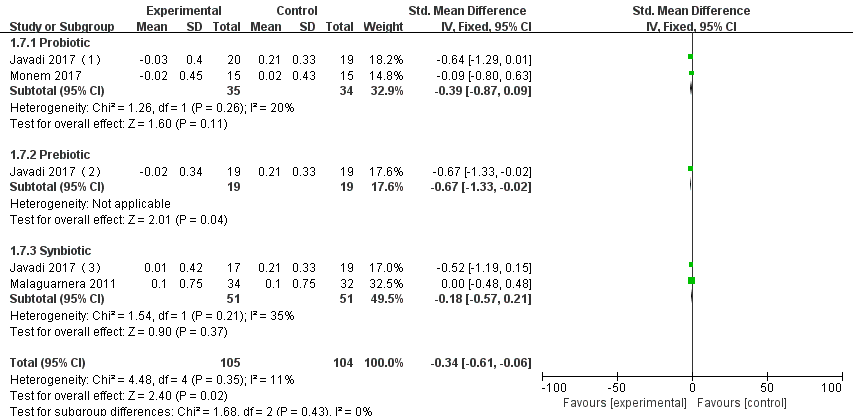


Figure S27.LDL-C-type of control Figure S28.ALB-type of intervention


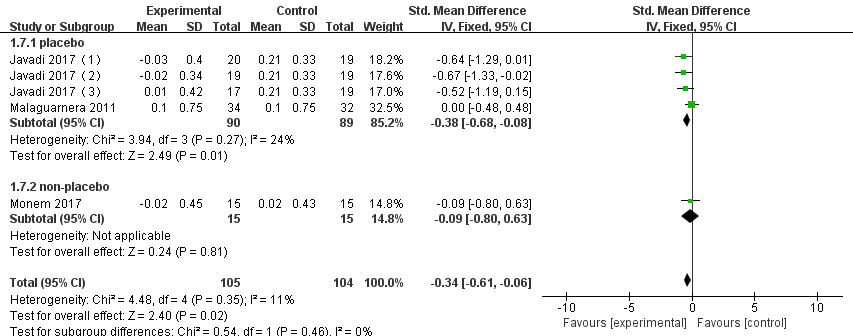


Figure S29.ALB-type of control

**
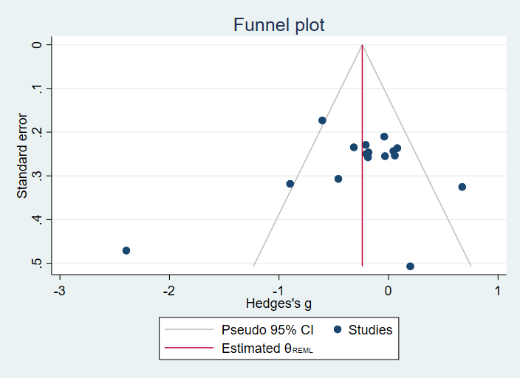

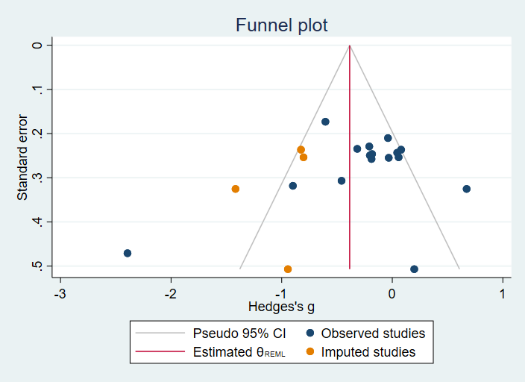
**

Figure S30. Publication bias of Glucose

**
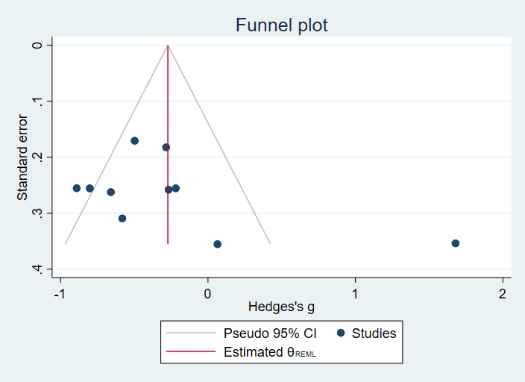

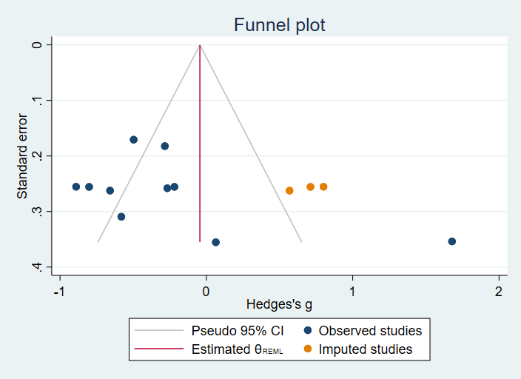
**

Figure S31. Publication bias of HOMA-IR


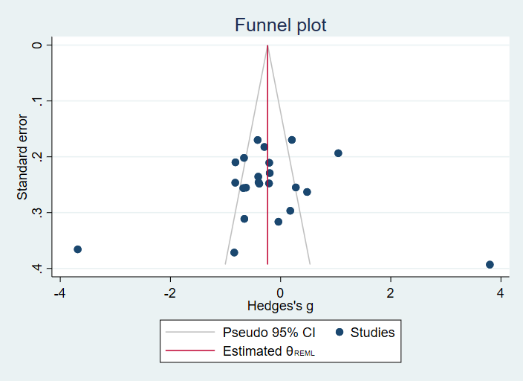

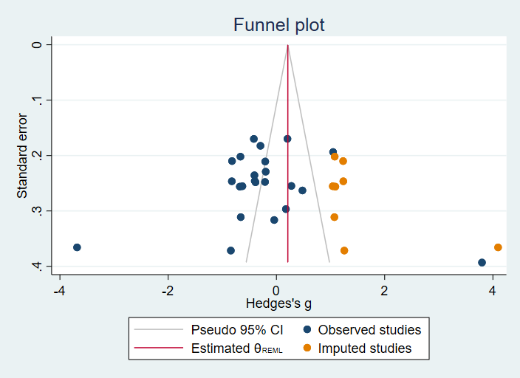


Figure S32. Publication bias of TG


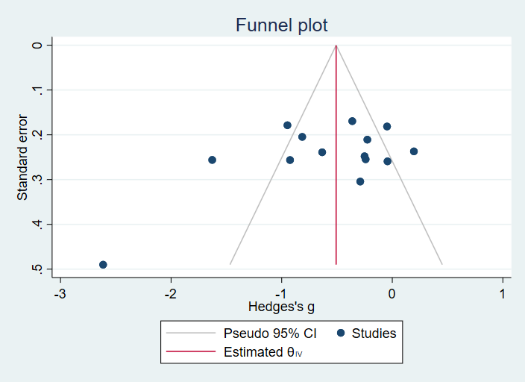

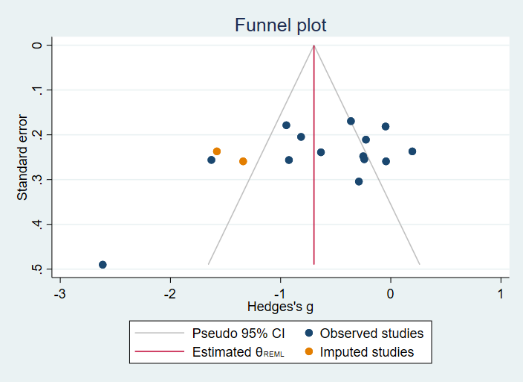


Figure S33. Publication bias of LDL-C


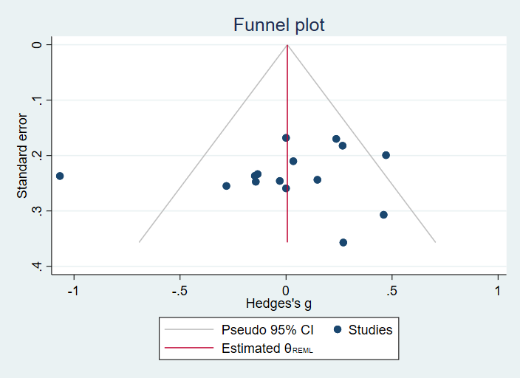

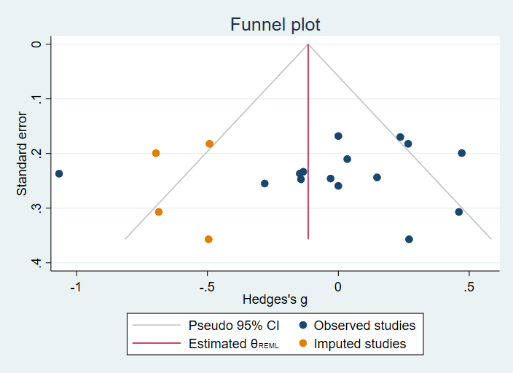


Figure S34. Publication bias of HDL-C

**
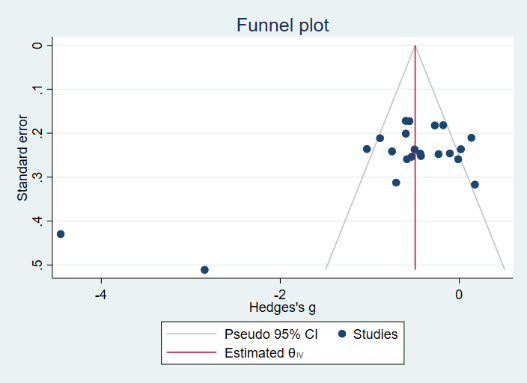
**

Figure S35. Publication bias of TC
